# Massive interstitial solid solution alloys achieve near-theoretical strength

**DOI:** 10.1038/s41467-022-28706-w

**Published:** 2022-03-01

**Authors:** Chang Liu, Wenjun Lu, Wenzhen Xia, Chaowei Du, Ziyuan Rao, James P. Best, Steffen Brinckmann, Jian Lu, Baptiste Gault, Gerhard Dehm, Ge Wu, Zhiming Li, Dierk Raabe

**Affiliations:** 1 Max-Planck-Institut für Eisenforschung, Max-Planck-Straße 1, 40237 Düsseldorf, Germany; 2 Department of Mechanical and Energy Engineering, Southern University of Science and Technology, Shenzhen, China; 3 School of Metallurgical Engineering, Anhui University of Technology, Maanshan, 243000 China; 4 Microstructure and Properties of Materials (IEK-2), Forschungszentrum Jülich, 52425 Jülich, Germany; 5 Department of Mechanical Engineering, City University of Hong Kong, Hong Kong, China; 6 Department of Materials, Royal School of Mine, Imperial College London, London, SW7 2AZ UK; 7 Center for Advancing Materials Performance from the Nanoscale (CAMP-Nano) and Hysitron Applied Research Center in China (HARCC), State Key Laboratory for Mechanical Behavior of Materials, Xi’an Jiaotong University, Xi’an, 710049 China; 8 School of Materials Science and Engineering, Central South University, Changsha, 410083 China; 9 Key Laboratory of Nonferrous Metal Materials Science and Engineering, Ministry of Education, Central South University, Changsha, 410083 China

**Keywords:** Engineering, Structural properties, Mechanical properties, Metals and alloys

## Abstract

Interstitials, e.g., C, N, and O, are attractive alloying elements as small atoms on interstitial sites create strong lattice distortions and hence substantially strengthen metals. However, brittle ceramics such as oxides and carbides usually form, instead of solid solutions, when the interstitial content exceeds a critical yet low value (e.g., 2 at.%). Here we introduce a class of massive interstitial solid solution (MISS) alloys by using a highly distorted substitutional host lattice, which enables solution of massive amounts of interstitials as an additional principal element class, without forming ceramic phases. For a TiNbZr-O-C-N MISS model system, the content of interstitial O reaches 12 at.%, with no oxides formed. The alloy reveals an ultrahigh compressive yield strength of 4.2 GPa, approaching the theoretical limit, and large deformability (65% strain) at ambient temperature, without localized shear deformation. The MISS concept thus offers a new avenue in the development of metallic materials with excellent mechanical properties.

## Abstract (web summary)

Interstitials can substantially strengthen metals. Here the authors show a massive interstitial solid solution (MISS) approach enabling a model multicomponent alloy to achieve near-theoretical strength together with large deformability.

## Introduction

The history of using mixtures of metallic elements to make stronger and tougher alloys exceeds 5 millennia^1^. In substitutional solid solution alloys, some of the solvent lattice atoms are replaced by solutes, and their size and electronic mismatch lead to distortions which impede the motion of dislocations, increasing the materials’ strength^2^. The recently developed high-entropy alloys, comprising complex concentrated solid solutions of multi-principal elements, benefit from massive substitutional solid solution strengthening and achieve in part excellent mechanical properties^3,4^. It is found that substitutional solutions approach their limits in further improving properties as the maximum lattice distortion attainable through substitutional atomic blending sets an upper bound^5^. Therefore, interstitials are attractive alternative alloying elements, as small atoms on interstitial sites create higher lattice distortions than substitutional ones, thereby substantially strengthening metals^6^. The strong strengthening effect of interstitials is known for steels, i.e., Fe-C, where doping with only ~0.1 wt% of C into Fe increases the strength by ~300 MPa^2^. The strong interaction of interstitials with lattice defects such as dislocations^7^, grain boundaries^8^, and precipitates^9^ offers a variety of pathways for strengthening. Although interstitial alloys can have high strength, it has long been challenging to achieve the theoretical strength limit of around *G*/10 (*G* is the material’s shear modulus) as proposed by Frenkel nearly a century ago^10^. In general, the deformability of alloys decreases with increasing interstitial contents^2^. Also, brittle ceramics such as oxides and carbides usually form, instead of solid solutions, when the interstitial content exceeds a critical yet low value (e.g., 2 at%)^11^, dramatically decreasing the alloys’ deformability^12^.

Here we present a solution to overcome the above challenges via introducing a concept of massive interstitial solid solution (MISS) alloys. The MISS alloy concept is realized by using a concentrated body-centered cubic (bcc) substitutional solid solution as highly distorted host matrix, which allows introducing massive amounts of interstitials as an additional principal element class. The atomic size differences in the concentrated substitutional solid solution create a wide distribution of expanded and compressed interstitial sites^13^, capable of solving massive amounts of interstitials. It also breaks the symmetry of the interstitial sites, thus counteracting the formation of long-range ordered oxides/carbides^14^. The massive interstitial content also reduces the free mixing enthalpy of the alloy system^15^, contributing to solid-solution stability. Thus, the MISS alloy concept overturns the previous alloy design strategies which are based on either negligible or small interstitial doping. More specifically, we define MISS alloys as solid solutions containing more than 5 at% interstitials. For a bcc TiNbZr-O-C-N MISS model system, we show here that the content of interstitial O reaches 12 at%, and no ceramic phases are formed. The alloy has an ultrahigh yield strength approaching the theoretical limit (*G*/18) and large deformability (65% strain) in compression at ambient temperature, without localized shear deformation. The MISS concept thus offers a next step in the design of metallic materials with excellent mechanical properties, by enabling the use of abundant and low-cost interstitial alloy ingredients.

## Results

### Structural and elemental information about the MISS alloys

**Fig. 1:**
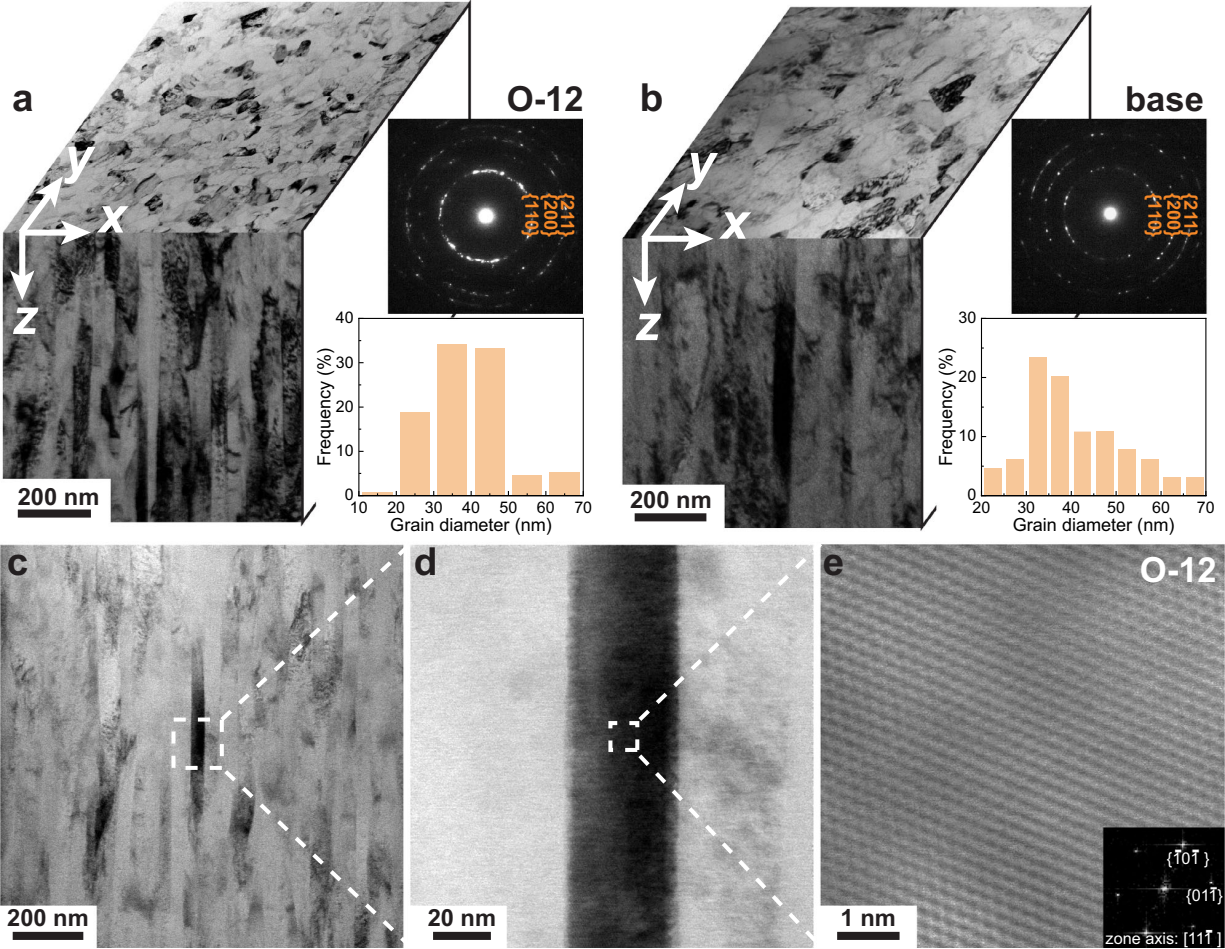
Microstructures of the (TiNbZr)_86_O_12_C_1_N_1_ (O-12) MISS alloy and the equiatomic TiNbZr (base) alloy. Typical side-view and in-plane-view TEM images of **a** the O-12 MISS alloy and **b** the interstitial-free base alloy, which serves here as a reference material. The *x-y* plane denotes the surface of the alloy and the *z* axis indicates the depth from the surface. The insets in **a** and **b** are the corresponding SAED patterns taken from side-view TEM specimens (top-right) and the distribution of the columnar grain diameters (bottom-right). **c, d, e** Annular bright-field scanning transmission electron microscopy (ABF-STEM) images of the O-12 MISS alloy, showing bcc structure without oxides, carbides, or nitrides. The inset in **e** is the corresponding fast Fourier transformation (FFT) pattern.

We realized the concept of MISS alloys by introducing high amounts of O (12 at%), C (~1 at%) and N (~1 at%) into a highly concentrated equiatomic TiNbZr solid solution alloy which serves here as host matrix (also referred to as medium entropy alloy in the literature^16^). The material with composition (TiNbZr)_86_O_12_C_1_N_1_ (at%), hereafter referred to as O-12 MISS alloy, was prepared through magnetron sputtering and subsequent annealing at 500 °C (“Methods”). The content of O reaches 12 at%, which is approaching the solubility limit for this element in the current alloy systems, i.e., 4 at% higher than that in bcc Ti-O (1730 °C), 6 at% higher than that in bcc Nb-O (1930 °C), and similar as that in bcc Zr-O (1970 °C) (Supplementary Fig. 1a). The C and N contents also approach the solubility limit of the TiNbZr alloy, i.e., ~1 at% in the TiNbZr-C system and ~1 at% in the TiNbZr-N system, respectively, according to the calculated phase diagrams (Supplementary Fig. 1b, c). For comparison, four reference alloys were fabricated, i.e., equiatomic TiNbZr as a crystalline base and reference alloy, a (TiNbZr)_92_O_6_C_1_N_1_ (O-6) crystalline MISS alloy, a (TiNbZr)_92_O_8_ (O-8) crystalline MISS alloy, and a (TiNbZr)_92_O_6_C_1_N_1_ (O-6A) amorphous alloy. The amorphous structure of the O-6A alloy is formed due to the enhanced glass forming ability achieved by introducing O and C^15^ in conjunction with a high cooling rate^17^ (above 10^8 ^K/s) during fabrication. The amorphous structure of the O-6A alloy is revealed by transmission electron microscopy (TEM, Supplementary Fig. 2a). TEM observations indicate that the O-6 (Supplementary Fig. 2b), O-12 (Fig. 1a), and base (Fig. 1b) alloys have bcc structure and consist of nanocolumnar grains with comparable diameters mainly ranging from 20 to 50 nm. No oxides, carbides, or nitrides are observed in the O-12 MISS alloy (Fig. 1c–e), consistent with the results from the selected area electron diffraction analysis.

**Fig. 2:**
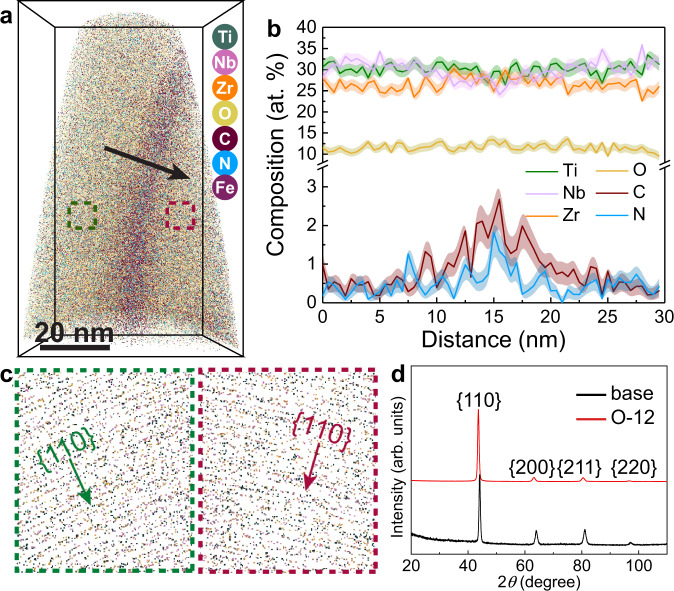
APT and XRD characterization of the O-12 MISS alloy. **a** Side-view of the three-dimensional reconstruction of a typical APT dataset. **b** 1D compositional profile measured along the length direction of the arrow displayed in **a**, showing that C and N are enriched at the grain boundary. **c** Thin slice through the APT dataset revealing two sets of {110} atomic planes. The lattice spacing is 0.24 nm. The positions of the two families of {110} planes are indicated by the rectangles in **a**. **d** XRD patterns of the O-12 and base alloys, presenting single-phase bcc structure.

The near-atomic scale chemical compositions of the TiNbZr-O-C-N MISS alloys were characterized by using atom probe tomography (APT). Figure 2 presents the APT results of the O-12 MISS alloy. The average composition of this alloy is Ti_30.7_Nb_30.2_Zr_25.9_O_11.6_C_0.8_N_0.5_Fe_0.3_ (at%). A detailed examination of the APT dataset shows two families of {110} atomic planes (Fig. 2a, c). These atomic planes revealed by APT exhibit an interplanar spacing of 0.24 nm, in good correspondence with the {110} planes identified by STEM (Supplementary Fig. 3). The two sets of {110} planes show a misorientation angle of around 37°, representing two abutting grains separated by a high-angle grain boundary. The 1D compositional profile in Fig. 2b indicates that C and N prominently segregate to the grain boundary, with concentrations of 1.8 ± 0.3 at% and 1.0 ± 0.2 at%, respectively, following the Gibbs adsorption isotherm^18^ (see “Methods”). The solubilities of the constituents in an alloy determine their segregation tendency to grain boundaries^19^, i.e., the low bulk solubilities of C and N entail a high grain boundary enrichment tendency. In contrast, Ti, Nb, and Zr have a strong affinity for O, and O shows a low tendency for segregation to grain boundaries^20^. The projected atom maps of O, C, and N within the grain boundary plane reveal a homogeneous distribution of these elements (Supplementary Fig. 4). The statistical frequency distribution analysis shows that all elements are uniformly distributed inside the nanograins (Supplementary Fig. 5), on the scale accessible to APT^21^. The APT analysis thus confirms the absence of oxides, carbides, or nitrides at grain boundaries and inside the grains, supporting the TEM findings. Also, the X-ray diffraction (XRD) patterns (Fig. 2d) present a single-phase bcc structure in both the O-12 and base alloys. The massive amounts of interstitials increase the lattice parameter from 3.376 Å of the base alloy to 3.402 Å of the O-12 alloy. These results confirm that a massive interstitial content has been successfully incorporated into the bcc lattice of the TiNbZr-O-C-N MISS alloys without any phase separation. As a reference material with lower interstitial content, the O-6 MISS alloy also shows C and N enrichment at the grain boundaries (Supplementary Fig. 6). The O-8 and amorphous O-6A alloys exhibit homogeneous elemental distributions when probed at near-atomic scale (Supplementary Fig. 7a and b). Furthermore, the base material, an equiatomic TiNbZr alloy, has negligible O, C, or N content, confirming that neither the sputtering vacuum conditions nor the interstitial-free alloy target introduce O, C, or N into the materials (Supplementary Fig. 7c).

### Mechanical properties

**Fig. 3:**
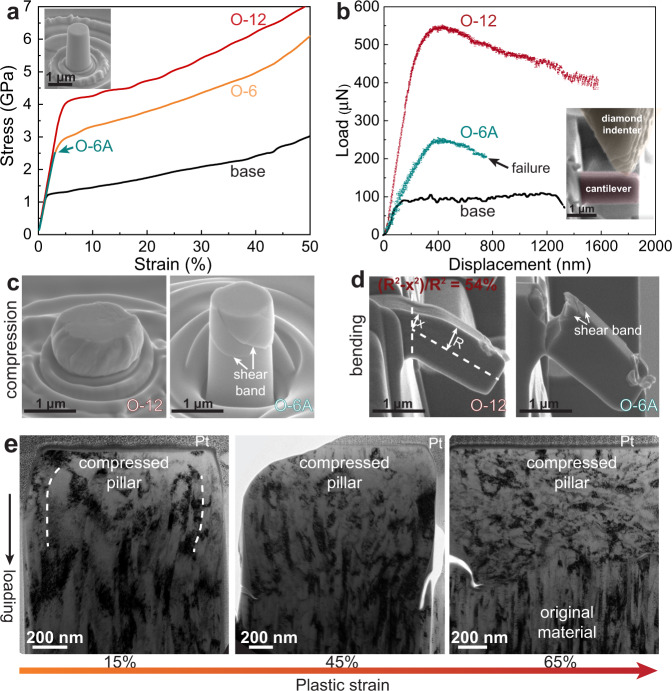
Mechanical properties of the TiNbZr-O-C-N MISS alloys and the reference equiatomic TiNbZr base alloy. **a** Compressive engineering stress-strain curves of the base, O-6, O-6A, and O-12 alloys measured at ambient temperature. The curves are shown only up to 50% strain to clearly reveal the yield points (the compressive deformation of the O-6 and O-12 alloys is even higher, above a total strain of 65%). The inset in **a** shows the O-12 MISS alloy pillar prior to compression. The arrow in **a** indicates the onset of strain bursts in the amorphous O-6A alloy, due to shear banding, leading to early failure. **b** Load-displacement curves measured by bending of cantilever beams, fabricated from the base, O-6A, and O-12 alloys. The inset in **b** shows the position of the diamond indenter tool, deforming the O-12 cantilever. SEM images presenting **c** micro-pillars and **d** cantilevers of the O-12 and O-6A alloys after deformation. Some shear bands in the deformed O-6A amorphous alloy are indicated by the arrows in **c** and **d**, respectively. **e** TEM images revealing the grain structure evolution of the O-6 MISS alloy with increasing compressive strain. After ~15% strain, the originally columnar grains have become curved. As the strain increases to an intermediate deformation state of ~45%, both, curved columnar grains and newly formed sub-grains are found. With further increasing the strain to ~65%, the initially columnar nanograins have become globular.

The mechanical properties of the TiNbZr-O-C-N MISS and TiNbZr alloys were evaluated via uniaxial compression experiments under identical conditions, e.g., same micropillar size and strain rate (Fig. 3a and c). The elastic moduli (*E*) of the alloys extracted from the stress-strain curves agree well with the values obtained from nanoindentation, which is 95 GPa for the O-12 MISS alloy and 85 GPa for the others. The base alloy exhibits a compressive yield strength (*σ*_*y*_) of 1.3 ± 0.1 GPa and continuous plastic flow at ambient temperature. The yield strength is 33% higher than that of a TiNbZr reference alloy with its millimeter sized grains^22^, which is expected considering the Hall–Petch effect^23,24^. The amorphous O-6A alloy has a yield strength of 2.5 ± 0.1 GPa, but the strain shows deformation bursts immediately after yielding, representing the strong yet brittle signature of metallic glass^25^. Shear bands are observed on the surface of the deformed O-6A amorphous alloy (Fig. 3c), reflecting the typical deformation behavior of metallic glasses. The nanograined O-6 MISS alloy shows a high yield strength of 2.9 ± 0.2 GPa, and large deformability to 65% strain in compression. The yield strength is nearly three times that of the base alloy and even exceeds the strength of its metallic glass counterpart. Strikingly, with further increasing interstitial content to 14 at%, the O-12 MISS alloy achieves an even significantly higher compressive yield strength of 4.2 ± 0.2 GPa, still maintaining a very high deformability (65% strain) at ambient temperature, without localized shear deformation. The shear stress for the initiation of plastic flow (*τ*_*y*_) can be converted using *τ*_*y*_ = *σ*_*y*_/2, a relationship suggested for compression experiments on alloys^26^. The shear modulus (*G*) of O-12 MISS alloy is calculated to be 38 GPa based on the experimentally measured elastic modulus. Notably, the *τ*_*y*_/*G* ratio of the O-12 MISS alloy reaches 1/18, which surpasses those of conventional nanograined alloys (*τ*_*y*_/*G~*1/100) and approaches the theoretical shear strength limit^10^ (*τ*_*y*_/*G~*1/10).

A sample size effect on the mechanical properties has been observed when the dimension of the material becomes comparable to the correlation length of the defects that serve as plastic deformation carriers, which is often manifested as “smaller is stronger”^27^. We tested O-12 pillars with diameters ranging from 1.0 to 1.6 μm, and the strength values were found to not change (Supplementary Fig. 8). In the O-12 MISS alloy, the structural unit (columnar grain) has a diameter of ~40 nm. This means that a 1.0 μm-diameter pillar contains hundreds of columnar grains, and thus multiple internal defects such as grain boundaries and dislocations. Considering that the sample size-reduction induced strength increase mostly originates from a low probability of containing defects or defect sources in small sized samples^28^, the sample size effect is thus not a relevant feature in the present study. It is noted that the use of a Ga^+^ ion beam for specimen preparation could in some cases alter the composition and structure of *~*10-nm-thick surface layers of alloys, an effect which may affect their mechanical response^29^. To minimize this effect, the milling current was therefore controlled to be as small as possible (1.5 pA for the last milling step). For comparison we also prepared O-12 micropillars using a Xe^+^ ion beam with negligible ion-irradiation and insignificant alloying effect^30^. The pillars fabricated using Ga^+^ and Xe^+^ ion beam milling show consistent ultrahigh yield strength and deformability (Supplementary Fig. 9). Therefore, we conclude that the O-12 MISS alloy achieves a near-theoretical compressive strength and high plastic formability.

In order to investigate if the MISS alloy shows also such excellent deformability under tensile load, we investigated the strength and plastic response of the material by using two types of additional tests, namely bending and tensile testing. In the in-situ SEM bending approach, the cantilevers that are exposed to bending, experience a tension state in the upper region and a compression state on the opposite side (Fig. 3b and d). For comparison and as a reference, the base alloy and the O-6A amorphous alloy were also tested under bending conditions, showing maximum flow stresses of 1.8 ± 0.3 GPa and 3.1 ± 0.3 GPa, respectively. The base alloy reveals necking upon bending (Supplementary Fig. 10), while the O-6A amorphous alloy fractured rapidly, showing formation of shear bands and cracks (Fig. 3d). In contrast, the O-12 MISS alloy exhibits an ultrahigh flow stress of 5.6 ± 0.5 GPa at ambient temperature, which is more than three times that of the base alloy. The O-12 MISS alloy reveals a dramatically higher flow stress compared to that of the O-6A and base alloys (tested under identical bending conditions), thus corroborating the results obtained from the compression tests and validating the significant strengthening effect achieved by the massive interstitial solid solution approach. The upper portion of the O-12 cantilever beam withstands a high tensile strain of 54% (quantified as a percent reduction in the necking area). No cracking or slip band formation was observed in the deformed cantilever, confirming the high plastic formability of the O-12 MISS alloy. This applies particularly to the upper surface of the bent cantilever which experienced tensile load.

In addition to these bending tests, we conducted uniaxial tensile tests using an in-situ SEM tension test protocol (Supplementary Fig. 11). Under these in-situ SEM tensile conditions, the O-12 MISS alloy exhibits a high ultimate tensile strength and plastic necking, underpinning its ductile response even for such a high strength level and under tensile conditions^31^. We also performed additional compression tests on the O-12 alloy using the same loading direction as used for the tensile tests, i.e., loading the material perpendicular to the columnar grain boundaries (Supplementary Fig. 12). We note that the mechanical properties of the O-12 alloy along the transverse direction are also excellent, with a high compressive yield strength of 3.4 GPa and large deformation to a high strain (12%). The lower yield strength compared to that tested in the longitudinal direction (4.2 GPa) may result from grain boundary activities in the transverse direction.

### Structure evolution upon plastic deformation

**Fig. 4:**
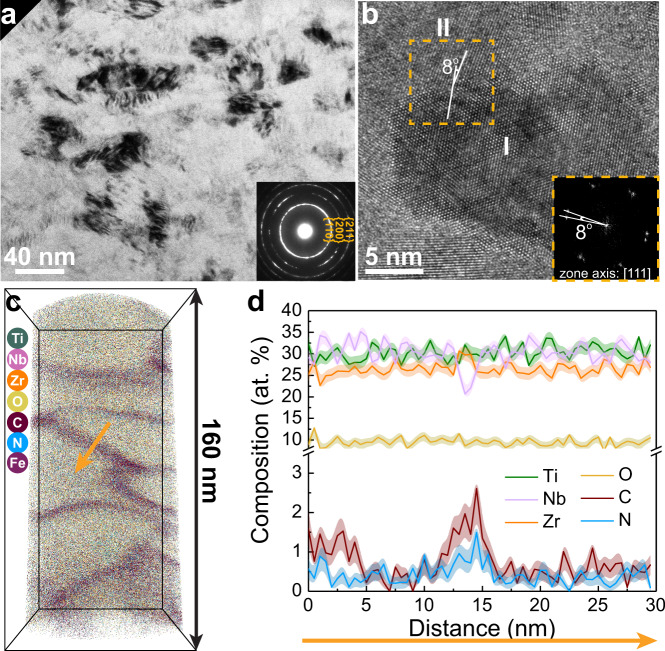
Deformation mechanisms of the O-12 MISS alloy. **a** A side-view TEM image of the O-12 MISS alloy upon 65% compressive strain, showing that the grains have been refined. The inset image is the corresponding SAED pattern. **b** A representative HRTEM image of the deformed structure containing two grains (labeled as I and II) observed along the [111] zone axis. The inset is the FFT pattern of the rectangle region in **b**, indicating an 8° misorientation between the two abutting grains. **c** Three-dimensional reconstruction of a typical APT specimen taken from the deformed material, revealing the grain boundary shape change after deformation. **d** 1D compositional profile measured by APT along the arrow displayed in **c**. The composition of the grain boundaries is similar to those in the as-fabricated O-12 MISS alloy.

To understand the deformation mechanisms of the TiNbZr-O-C-N MISS alloys, the structure evolution upon deformation was systematically investigated with TEM and APT. The compressed pillars in O-6 MISS alloy show a barreled geometry without formation of shear/slip band at varied strains, confirming the high plastic formability of the alloy (Fig. 3e and Supplementary Fig. 13). At a strain of ~65%, the original columnar grains have become globular, indicating occurrence of deformation-driven grain refinement. The O-12 MISS alloy reveals a similar deformation feature. Grains with initial diameters of 20–50 nm have been refined to diameters ranging from 5 nm to 20 nm upon deformation to a strain of ~65% (Fig. 4a). Representative high-resolution TEM observation conducted along the [111] zone axis shows that one of the closely packed {111} planes of grain I is inclined by 8° relative to that of grain II (Fig. 4b). The fast Fourier transformation image confirms that an 8° misorientation exists between the two adjacent grains. We further investigated the near-atomic scale elemental distribution in the compressed O-12 alloy pillar using APT. The grain boundaries appear arc-shaped and consist of 1.7 ± 0.4 at% C and 0.9 ± 0.2 at% N, showing a composition similar to those in the as-fabricated O-12 MISS alloy (Fig. 4c, d). The APT investigation reveals the shape and distribution of the grain boundaries of the deformed O-12 alloy, which matches the TEM observation and confirms the occurrence of grain refinement during deformation. Conventional nanograins are known to be extremely unstable under stress and grain growth induced softening is often observed^32^. The absence of grain growth in the O-6 and O-12 MISS alloys indicates that the grain boundaries in these alloys are stable against stress and capillary-driven coarsening, i.e., deformation driven grain boundary migration is suppressed, so that no grain-growth related softening occurs.

## Discussion

We note that the complex local chemical bonding structures in highly concentrated solid solutions may promote short-range order^33–35^ or concentration fluctuation^36^ in certain alloys. Here, the O-12 MISS alloy reveals a homogeneous distribution of the elements, down to the near-atomic scale in the grain interiors (Supplementary Fig. 5), indicating that the distorted lattices are uniformly distributed in the alloy. The interstitials occupy the octahedral interstitial sites in the bcc lattice structure, which causes asymmetrical lattice distortion and creates strong force fields, impeding dislocation nucleation and motion^37^. In the MISS alloys, the interstitials can serve as pining sites for the kink-pairs, resulting in an increased activation energy required to overcome the saddle-point configuration for percolating dislocation motion. This pinning of the kink-pairs increases the friction stress for dislocation motion. The friction associated with the secondary Peierls potential is also increased, which impedes the movement of the kinks along the dislocation line. In conventional nanograined alloys, grain boundaries can act as dislocation sources^38^. In the O-12 MISS alloy, however, the grain boundaries are stabilized by C and N, leading to higher stresses for dislocation nucleation. We evaluated the strength increase due to interstitials using Labusch’s method^39^, and confirmed that the strengthening effect is mostly ascribed to the O interstitials, accompanied by C and N segregation at grain boundaries (see “Methods”).

In conventional interstitial O-doped alloys, dislocations are usually confined to a limited set of preferred glide planes^40^. The high density of dislocations accumulating on them thus leads to pile-up configurations resulting in stress concentrations near interfaces, facilitating localized deformation^37^. The O-8 MISS alloy without grain boundary segregation reflects this trend, showing enhanced planar slip with reduced deformability, although no oxides are present (Supplementary Fig. 14). In the O-12 MISS alloy, containing even more O interstitials, however, the C and N segregation to grain boundaries reduces the interface energy^41^. The stabilized grain boundaries reveal enhanced resistance to the transmission of slip from one grain to another^42^. This mechanism counteracts slip localization in the O-12 MISS alloy, thus preventing the localized deformation which is observed in the O-doped alloy without grain boundary segregation (Supplementary Fig. 14). The intense dislocation intersection and multiplication in alloys upon deformation can simultaneously enhance their strength and ductility^43^, while the same effect is usually challenging to achieve in nanograined alloys, owing to the instability of their grain boundaries^44^. Here, the segregation of C and N stabilizes the grain boundaries of the O-12 MISS alloy. The dislocations are confined in the nanograins of the O-12 alloy, in which the strongly reduced mean free path of dislocations enhances the rates of dislocation multiplication. This promotes sub-grain boundary formation at the interfaces as dislocations are accumulated there, thus further reducing the mean free path of dislocations. With increasing strain, nanograins that are formed by dislocation-driven grain subdivision would be expected to undergo grain growth or grain boundary sliding, both of which would entail mechanical softening^32^. However, such activity is suppressed in the current O-6 and O-12 MISS alloys as C and N decorate and stabilize the grain boundaries through the reduction of their interfacial energy^45^. To evaluate the effect of grain boundary segregation on the interface stability, the base and O-6 alloys were annealed at 500 °C for 2 h (Supplementary Fig. 15). The base alloy experiences grain growth from ~40 to ~200 nm, accompanied by a reduction of its strength. In contrast, the O-6 alloy reveals unchanged grain size and also unchanged mechanical properties after annealing. This finding confirms that C and N segregation reduces the grain boundary energy and thus enables high grain boundary stability. Grain boundary stabilization through relaxation and solute segregation prevents grain boundary motion and results in hardening of the extremely fine grained materials^42^. As a result, the newly formed grain boundaries remain stable and further impede dislocation motion upon loading, which contributes to mechanical stability also at larger strains.

In summary, we demonstrate a counterintuitive alloy design approach which consists in blending a massive interstitial content as an additional set of principal elements into a distorted host matrix instead of using only the usual minor interstitial doping. This alloy class is referred to as MISS. The representative (TiNbZr)_86_O_12_C_1_N_1_ (at%) MISS alloy achieves an ultrahigh yield strength of 4.2 GPa in compression, approaching the theoretical strength limit of ~*G*/10. The near-theoretical compressive strength is mostly ascribed to the high amount of O interstitials (12 at%) that strongly impede dislocation nucleation and motion. The O interstitials promote dislocation multiplication at high stress levels, while the C and N segregation to grain boundaries suppresses the localized deformation. These two effects promote deformation-driven grain refinement and enable high deformability (65% strain without localized shear deformation) under micropillar compression conditions. This alloy design strategy is applicable to a wide range of materials including alloys consisting of multiple principal elements, and may provide fundamental insights into the nature of plastic deformation in massive interstitially doped solid solutions.

## Methods

### Phase diagram calculations

The phase diagrams of TiNbZr-C and TiNbZr-N systems were calculated using the Thermo-Calc software in conjunction with the TCHEA4 database. Due to the lack of the important interstitial element O in that database, the solubility limit of O in the current TiNbZr host alloy could only be assessed and approximated by calculating the binary phase diagrams of Ti-O, Nb-O, and Zr-O systems using the TCBIN1 database.

### Design of the MISS alloys

The phase diagram calculations indicate that the solubility limit of O is 8 at% in bcc Ti at 1730 °C, 6 at% in bcc Nb at 1930 °C, and 12 at% in bcc Zr at 1970 °C, respectively (Supplementary Fig. 1). We note that thermodynamic calculations cannot yet fully consider all relevant aspects here such as lattice distortions and the variation in interstitial sites of concentrated substitutional solid solutions, however, the first calculations suggest that the lower solubility limit of O is 6 at.% in the Ti-O, Nb-O, and Zr-O systems. We also considered the definition of multi-principal-element alloys, i.e., alloys containing multiple substitutional principal elements in relatively high concentrations from 5 to 35 at%^16^. Based on our thermodynamic modeling and the widely adopted multi-principal-element alloy concept, we suggest a lower limit of 5 at% interstitials for MISS alloys. We thus prepared the O-6 MISS alloy containing 6 at% of O, and further increased the interstitial O content to approach the possible upper solubility limit of 12 at% (O-12 MISS alloy).

In addition to the massive amounts of O interstitials inside the nanograins, C and N interstitials were also introduced to achieve grain boundary segregation, driven by the Gibbs adsorption isotherm:^18^ $d\gamma =-{\sum }_{i}{\Gamma }_{i}d{u}_{i}$, where *γ* is the grain boundary energy, *Γ*_*i*_ is the grain boundary excess concentration of the element *i*, and *u*_*i*_ is the chemical potential. This isotherm^18^ states that strongly segregating elements leads to a reduction in grain boundary energy. If we only use one element (C or N) to decorate grain boundaries, its content should be higher than 1 at% to ensure a similar effect of grain boundary decoration and stabilization compared to the O-6 ((TiNbZr)_92_O_6_C_1_N_1_ at%) and O-12 ((TiNbZr)_86_O_12_C_1_N_1_ at%) MISS alloys. However, a higher C or N content may promote heterogeneous nucleation and subsequent growth of carbides or nitrides, particularly in grain boundary regions due to the C and N enrichment. This may induce grain boundary embrittlement. Therefore, we simultaneously introduced C and N into the O-6 and O-12 MISS alloys, thus achieving both, highly interstitially alloyed nanograins and C-N stabilized grain boundaries.

### Materials preparation

The equiatomic TiNbZr base alloy, crystalline O-6 MISS alloy, and amorphous O-6A alloy films were deposited on Si (001) substrates using magnetron sputtering with a background vacuum below 10^-5 ^Pa. During sputtering, the Ar pressure was 0.3 Pa and the substrate bias power was 90 W. The deposition rate was kept 12 nm/min by controlling target power, and the film thickness is 4 μm. The TiNbZr base alloy films were produced by using an equiatomic TiNbZr alloy target fabricated using induction melting. The O-6 and O-6A alloys were fabricated by utilizing an alloy target produced by powder compaction, the O, C, and N were intentionally introduced through target preparation. The powders have large surface-to-volume ratios, enabling the uptake of light elements from the surfaces. The interstices between the powders can also serve as the reservoir of light elements such as O, C, and N during powder compaction. The equiatomic TiNbZr base alloy was fabricated at room temperature. The crystalline O-6 alloy was prepared at a substrate temperature of 400 °C, and its amorphous counterpart O-6A alloy was obtained at a substrate temperature of below 50 °C. The crystalline O-12 and O-8 MISS alloys were prepared by annealing the O-6 alloy and TiNbZr alloy in a vacuum (10^−2 ^Pa) at 500 °C for 2 h, respectively. These annealing conditions were selected to promote the diffusion of O interstitials into the alloy and to avoid the possible formation of oxides. The diffusion length (*L*) of O in elemental Ti, Nb, and Zr metals at 500 °C can be calculated using $L=\sqrt{(D\times t)}$, where *D* is the diffusivity and *t* is the diffusion time. The diffusivity of O, in a comparable vacuum as used in the present work for synthesis and heat treatment, through the hexagonal close-packed (hcp) structure of elemental Ti and Zr is similar, i.e., ~7 × 10^−13^ cm^2^/s at 500 °C^46^, corresponding to a value of *L* of ~1 μm over a duration of 2 h. The diffusivity of O in the bcc-structure of elemental Nb is ~5 × 10^−10^ cm^2^/s at 500 °C^47^ and leads to *L* of ~19 μm in 2 h. The much higher diffusivity in bcc metals compared to hcp is related to its non-close-packed lattice (only 68% volume filling vs. 74% volume filling of a near-close packed lattice), which promotes interstitial diffusion^2^. The TiNbZr alloys have a single-phase bcc structure which can promote a high interstitial diffusivity. Hence, the 2-h annealing at 500 °C in a vacuum (10^−2 ^Pa) allows for O to diffuse throughout the 4-μm-thick TiNbZr alloy films.

We note that the C and N contents in the grain boundaries of the O-12 alloy (1.8 ± 0.3 at% C and 1.0 ± 0.2 at% N) are slightly higher than that of the O-6 alloy (1.6 ± 0.5 at% C and 0.8 ± 0.3 at% N). The annealing process allows for solute diffusion to the grain boundaries, driven by the Gibbs adsorption isotherm^18^. The annealing-enhanced solute segregation to grain boundaries contributes further to a reduction of the interfacial energy, thus improving grain boundary stability^42^. Although the alloys in the present work were fabricated using magnetron sputtering and have micrometer thickness, the strengthening and plastic deformation mechanisms are generally applicable for bulk metallic materials. This MISS alloy strategy is especially useful for bcc structured alloys in which interstitials cause asymmetrical lattice distortion and create strong force fields that impede dislocation nucleation and motion.

### Evaluation of interfacial stability

To investigate the effect of C and N segregation on grain boundary stability, the equiatomic TiNbZr base alloy and O-6 alloy were encapsulated in quartz tubes and annealed at 500 °C with Ar atmosphere for 2 h. The quartz tubes were evacuated using a double stage rotation pump in combination with a turbomolecular pump to reach a high vacuum (<10^−4 ^Pa) first, and then filled with Ar (99.999% purity, Argon 5.0) to 25 kPa, a Ti ribbon was placed in each tube.

### Structure characterization

The structures of the films were characterized using grazing incidence XRD (GIXRD) on an ISO-DEBYEFLEX 3003 equipment with a Cobalt *K*_α1_ source (*λ* = 0.179 nm). The incidence angle was 2° and the step size was 0.03°. The microstructures were characterized using TEM (JEOL 2200F) operated at 200 kV. The ABF-STEM images were recorded on a probe-corrected STEM/TEM (FEI Titan Themis 80-300, operated at 300 kV). The LAADF-STEM images were taken on an image-corrected STEM/TEM (FEI Titan Themis 80-300, operated at 300 kV). The lamellas for TEM observation were prepared using a focused ion beam workstation (FIB, FEI Helios Nanolab 600i). The lamellas were cleaned using 2 kV, 23 pA at the last stage to avoid beam damage.

### Elemental composition analysis at atomic scale

The elemental compositions of the alloys were characterized by APT using a Cameca LEAP^TM^ 5000XR instrument. APT samples were prepared in a FIB workstation by annular milling, where the current was gradually decreased with decreasing specimen diameter. The last milling parameters used were 5 kV and 41 pA, followed by 2 kV and 23 pA. APT data were collected in high-voltage pulsing mode at 15% pulse fraction and a detection rate of 0.2%, under a high vacuum of around 3 × 10^−11^ mbar, at a temperature of 60 K. The Cameca integrated visualization and analysis software IVAS 3.8.4 was used for data analysis and three-dimensional atom map reconstruction. The 1D compositional profiles were obtained from 5-nm-diameter cylinders with a bin width of 0.2 nm.

### Mechanical property testing

The compression experiments were conducted in a nanoindenter (Agilent G200) using a flat 20-μm-diameter diamond tip in load-control mode at ambient temperature. All the compression tests were performed at a nominal strain rate of 5 × 10^−3 ^s^−1^. The micropillars for compression tests were fabricated in a FIB workstation. To minimize FIB induced damage, the milling current was controlled to be as low as possible and the final current was 1.5 pA. In order to minimize the effect of pillar geometry, the micropillars were carefully controlled to possess an aspect ratio (pillar height/width) between 2 and 3, and a taper angle below 1.5°. The pillar diameter was determined at 20% pillar height to guarantee an accurate measurement of strength, i.e., avoid overestimation of strength due to taper angle. The elastic moduli of the alloys were determined using nanoindentation methods with a Berkovich tip. The indentation depth was kept within 10% of film thickness to avoid substrate effect. The hardness is 9.2 ± 0.4 GPa, 7.2 ± 0.2 GPa, 6.5 ± 0.2 GPa, and 5.0 ± 0.3 GPa for O-12, O-6, O-6A, and base alloy, respectively.

We performed in-situ SEM bending tests using an Asmec Unat II indenter in displacement-controlled mode inside a Zeiss Gemini500 SEM at ambient temperature, with a 2-μm-width and 100-nm-diameter diamond wedge and a loading rate of 5 nm/s. At least three samples were tested for each condition to ensure repeatability. The diamond indenter was checked using SEM after testing to ensure a consistent indenter geometry. In bending testing, the neutral plane of the cantilever can retard dislocation motion and lead to dislocation pile-ups^48^. The strain gradient and the inhomogeneous stress fields in the cantilever prevent a simple correlation of the flow stress measured in bending with that in compression testing. Therefore, the base alloy and the O-6A amorphous alloy were tested under bending conditions for comparison and as a reference in addition to the O-12 alloy. The maximum flow stress in bending was determined according to the linear-elastic bending theory:^49^ *σ*_*max*_ = 6 × (*F*_*max*_ × *l*)/(*B* × *t*^*2*^), where *F*_*max*_ is the maximum load, *l* is the distance between the loading point and the fixed end of the cantilever, *B* is the width and *t* is the thickness of the cantilever. The fixed end of the cantilever withstands tensile deformation on the upper surface and compression on the opposite surface. The tensile strain was calculated in percent reduction in the area of the tensile side using: *ε* = (*R*^*2*^ − *x*^*2*^)/*R*^*2*^, where *R*^*2*^ is the cross-sectional area of the original cantilever and *x* is measured from the narrowest cross-section of the bent cantilever (see Fig. 3d).

The in-situ SEM tension experiments were carried out using a Hysitron PI88 Picoindenter with a diamond gripper in displacement-controlled mode inside a Zeiss Gemini500 SEM at ambient temperature and a loading rate of 2 nm/s. The in-situ bending and tension samples were prepared using a FIB workstation. The cantilevers for bending have a width of 1 μm, a thickness of 1 μm, and a length of 2 μm. The in-situ tension samples have square cross-sections with gauge width of 1.1 μm and length of 2.3 μm. Three tensile specimens were prepared from the O-12 alloy using the FIB workstation and tested using the PI88 Picoindenter.

### Finite element method simulation (FEM)

We used the Abaqus Finite Element simulation package (Dassault Systèmes Simulia Corp.) to model the tensile deformation of the O-12 alloy. We conducted 2D plane stress simulations using quadrilateral elements with reduced integration. A linear elasticity constitutive model was used in the simulations, with a Young’s modulus of 95 GPa and Poisson’s ratio of 0.3. The simulated specimen before deformation has an overall length of 5.0 μm, a gauge section with 1.1 μm-width and 2.3 μm-length, and has a 0.1 μm-rounding at the transition region between the gauge section and grip area, to replicate the sample dimension in tension experiments.

### Calculation of interstitial strengthening

The effect of the massive amounts of interstitials on the bcc structure alloy was evaluated using Labusch’s method^39^. The strength increment (*∆τ*) due to interstitials can be estimated following the formulations in refs: ^39,50,51^. *∆τ* = *G* × *∆ϵ*^4/3^ × *c*^2/3^, where *G* is the shear modulus, *∆ϵ* is the lattice misfit parameter corresponding to the distortion induced by interstitials, *c* is the atomic content of the interstitials in solid solution. Here we use the change of hardness, *∆H* = 3^3/2^× (*G* × *∆ϵ*^4/3^ × *c*^2/3^), to quantify the solid solution strengthening effect. A factor of 3^3/2^ was used for conversion from shear stress to hardness^52^. The shear modulus of the O-12 MISS alloy was calculated to be 38 GPa, the *∆ϵ* was estimated to be 0.10 for interstitial O and 0.14 for interstitial N^33^. Interstitial C was reported to have a less strengthening effect in bcc alloys compared with O and N, here the *∆ϵ* for C was taken as 3/4 that for O^53^. Based on the above parameters, the hardness increase due to O, N, and C interstitials is 2.2 GPa, 0.7 GPa, and 0.3 GPa, respectively. The grain boundary segregation contributes to an increase of 1.0 GPa considering a total hardness increment of 4.2 GPa. Unlike traditional interstitial strengthening that embrittles metals, the MISS strategy enables extraordinary strength and deformability simultaneously to the representative bcc structure TiNbZr-O-C-N alloys.

## Supplementary information

Supplementary Information

## Data Availability

All relevant data supporting the findings of this study are contained in the paper and its Supplementary Information files. All other relevant data are available from the corresponding authors (G.W., Z.L., and D.R.) upon request.

## References

[1] Callister, W. D. & Rethwisch, D. G. *Materials Science and Engineering*. 5 (John Wiley & Sons NY, 2011).

[2] Campbell, F. C. *Elements of Metallurgy and Engineering Alloys*. (ASM International, Materials Park, OH 2008).

[3] Lee C (2020). Lattice distortion enhanced yield strength in a refractory high-entropy alloy. Adv. Mater..

[4] Miracle DB, Senkov ON (2017). A critical review of high entropy alloys and related concepts. Acta Mater..

[5] Oh HS (2019). Engineering atomic-level complexity in high-entropy and complex concentrated alloys. Nat. Commun..

[6] Suzuki T., Takeuchi S., Yoshinaga H. *Dislocation Dynamics and Plasticity*. (Springer, Berlin, 1991).

[7] He BB (2017). High dislocation density–induced large ductility in deformed and partitioned steels. Science.

[8] Gao J (2021). Facile route to bulk ultrafine-grain steels for high strength and ductility. Nature.

[9] Jiang S (2017). Ultrastrong steel via minimal lattice misfit and high-density nanoprecipitation. Nature.

[10] Frenkel J (1926). Zur Theorie der Elastizitätsgrenze und der Festigkeit kristallinischer Körper. Z. Phys..

[11] Briant C. L., Banerji S. K. *Embrittlement of Engineering Alloys* (Academic Press, New York, 1983).

[12] Liu Z, Welsch G (1988). Effects of oxygen and heat treatment on the mechanical properties of alpha and beta titanium alloys. Metall. Trans. A.

[13] Lu C (2016). Enhancing radiation tolerance by controlling defect mobility and migration pathways in multicomponent single-phase alloys. Nat. Commun..

[14] Zhang Y (2017). Atomic-level heterogeneity and defect dynamics in concentrated solid-solution alloys. Curr. Opin. Solid State Mater. Sci..

[15] Li HX, Gao JE, Jiao ZB, Wu Y, Lu ZP (2009). Glass-forming ability enhanced by proper additions of oxygen in a Fe-based bulk metallic glass. Appl. Phys. Lett..

[16] George EP, Raabe D, Ritchie RO (2019). High-entropy alloys. Nat. Rev. Mater..

[17] Ketov SV (2015). Nanostructured Zr-Pd metallic glass thin film for biochemical applications. Sci. Rep..

[18] Gibbs J. W. *The Collected Works of J. Willard Gibbs*, (Yale University Press, New Haven, CT, 1948), **1**.

[19] Raabe D (2014). Grain boundary segregation engineering in metallic alloys: a pathway to the design of interfaces. Curr. Opin. Solid State Mater. Sci..

[20] Zhu JH, Liu CT (2002). Intermediate-temperature mechanical properties of Ni-Si alloys: oxygen embrittlement and its remedies. Intermetallics.

[21] De Geuser F, Gault B (2020). Metrology of small particles and solute clusters by atom probe tomography. Acta Mater..

[22] Senkov ON, Rao S, Chaput KJ, Woodward C (2018). Compositional effect on microstructure and properties of NbTiZr-based complex concentrated alloys. Acta Mater..

[23] Petch NJ (1953). The cleavage strength of polycrystals. J. Iron Steel Inst..

[24] Hall EO (1951). The deformation and ageing of mild steel: III discussion of results. Proc. Phys. Soc. Sect. B.

[25] Wang WH (2012). The elastic properties, elastic models and elastic perspectives of metallic glasses. Prog. Mater. Sci..

[26] Johnson WL, Samwer K (2005). A universal criterion for plastic yielding of metallic glasses with a (T/T_g_)^2/3^ temperature dependence. Phys. Rev. Lett..

[27] Banerjee A (2018). Ultralarge elastic deformation of nanoscale diamond. Science.

[28] Shim S, Bei H, George EP, Pharr GM (2008). A different type of indentation size effect. Scr. Mater..

[29] Shim S, Bei H, Miller MK, Pharr GM, George EP (2009). Effects of focused ion beam milling on the compressive behavior of directionally solidified micropillars and the nanoindentation response of an electropolished surface. Acta Mater..

[30] Liu J (2020). Effect of ion irradiation introduced by focused ion-beam milling on the mechanical behaviour of sub-micron-sized samples. Sci. Rep..

[31] Pan J, Ivanov YP, Zhou WH, Li Y, Greer AL (2020). Strain-hardening and suppression of shear-banding in rejuvenated bulk metallic glass. Nature.

[32] Li Q (2018). High-strength nanotwinned Al alloys with 9R phase. Adv. Mater..

[33] Lei Z (2018). Enhanced strength and ductility in a high-entropy alloy via ordered oxygen complexes. Nature.

[34] Zhang R (2020). Short-range order and its impact on the CrCoNi medium-entropy alloy. Nature.

[35] Chen X (2021). Direct observation of chemical short-range order in a medium-entropy alloy. Nature.

[36] Ding Q (2019). Tuning element distribution, structure and properties by composition in high-entropy alloys. Nature.

[37] Yang P-J (2019). Mechanism of hardening and damage initiation in oxygen embrittlement of body-centred-cubic niobium. Acta Mater..

[38] Wang L (2014). Grain rotation mediated by grain boundary dislocations in nanocrystalline platinum. Nat. Commun..

[39] Labusch R (1970). A statistical theory of solid solution hardening. Phys. Status Solidi.

[40] Gray GT, Luetjering G, Williams JC (1990). The influence of oxygen on the structure, fracture, and fatigue crack propagation behavior of Ti-8.6 Wt Pct Al. Metall. Trans. A.

[41] Curry JF (2018). Achieving ultralow wear with stable nanocrystalline metals. Adv. Mater..

[42] Hu J, Shi YN, Sauvage X, Sha G, Lu K (2017). Grain boundary stability governs hardening and softening in extremely fine nanograined metals. Science.

[43] Juan CC (2016). Simultaneously increasing the strength and ductility of a refractory high-entropy alloy via grain refining. Mater. Lett..

[44] Kumar KS, Van Swygenhoven H, Suresh S (2003). Mechanical behavior of nanocrystalline metals and alloys. Acta Mater..

[45] Li Y (2014). Segregation stabilizes nanocrystalline bulk steel with near theoretical strength. Phys. Rev. Lett..

[46] Wu, H. Oxygen Diffusion Through Titanium and Other HCP Metals, thesis, University of Illinois at Urbana-Champaign, (2013).

[47] Perkins RA, Padgett RA (1977). Oxygen diffusion in niobium and NbZr alloys. Acta Met..

[48] Dehm G, Jaya BN, Raghavan R, Kirchlechner C (2018). Overview on micro- and nanomechanical testing: New insights in interface plasticity and fracture at small length scales. Acta Mater..

[49] Matoy K (2009). A comparative micro-cantilever study of the mechanical behavior of silicon based passivation films. Thin Solid Films.

[50] Pöhl C, Schatte J, Leitner H (2013). Solid solution hardening of molybdenum-hafnium alloys: experiments and modeling. Mater. Sci. Eng. A.

[51] Wu Z, Gao Y, Bei H (2016). Thermal activation mechanisms and Labusch-type strengthening analysis for a family of high-entropy and equiatomic solid-solution alloys. Acta Mater..

[52] Schuh CA, Nieh TG, Iwasaki H (2003). The effect of solid solution W additions on the mechanical properties of nanocrystalline Ni. Acta Mater..

[53] Zeyfang R, Conrad H (1971). Deformation dynamics of a b.c.c. titanium alloy (15.2 at% Mo) below 650 °K (~0.4 T_m_). Acta Met..

