# Supplementary material for: Massive interstitial solid solution alloys achieve near-theoretical strength
Source: Nat Commun. 2022 Mar 1;13:1102. doi: 10.1038/s41467-022-28706-w (PMC8888583; doi:10.1038/s41467-022-28706-w)
Supplement: Supplementary file 1 — Supplementary Information [file 41467_2022_28706_MOESM1_ESM.pdf]

**Supplementary Information for**

# Massive interstitial solid solution alloys achieve near-theoretical strength

Chang Liu, Wenjun Lu, Wenzhen Xia, Chaowei Du, Ziyuan Rao, James P. Best, Steffen Brinckmann, Jian Lu, Baptiste Gault, Gerhard Dehm, Ge Wu*, Zhiming Li*, Dierk Raabe*

Correspondence to: gewuxjtu@xjtu.edu.cn; zhiming.li@mpie.de; d.raabe@mpie.de

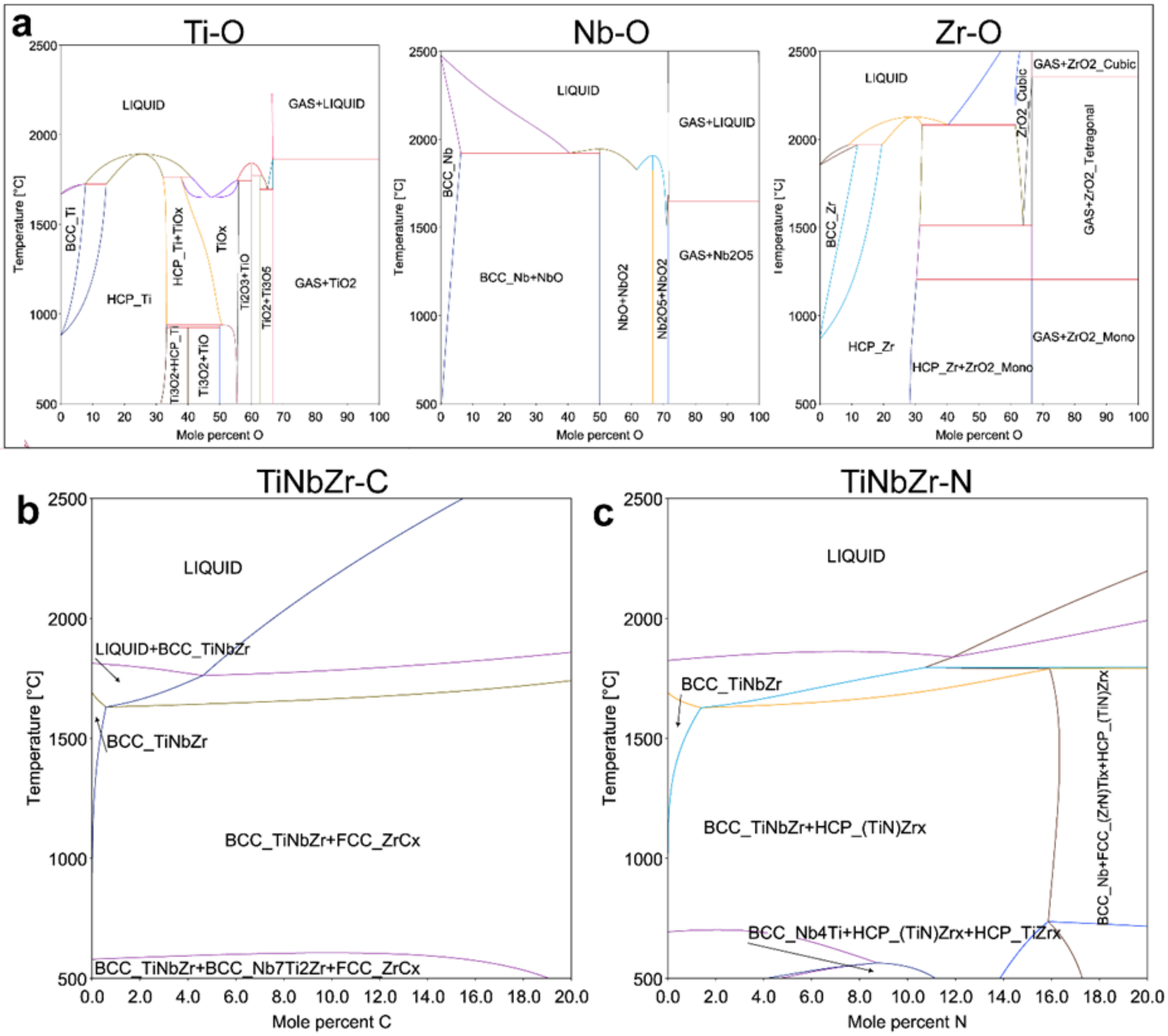


**Supplementary Fig. 1 Phase diagrams. a** Ti-O, Nb-O, and Zr-O phase diagrams. The solubility limit of O is 8 at.% in bcc Ti at 1730 °C, 6 at.% in bcc Nb at 1930 °C, 12 at.% in bcc Zr at 1970 °C, respectively. **b** TiNbZr-C phase diagram. The solubility limit of C is ~1 at.% in the TiNbZr-C system at 1630 °C. **c** TiNbZr-N phase diagram. The solubility limit of N is ~1 at.% in the TiNbZr-N system at 1630 °C.

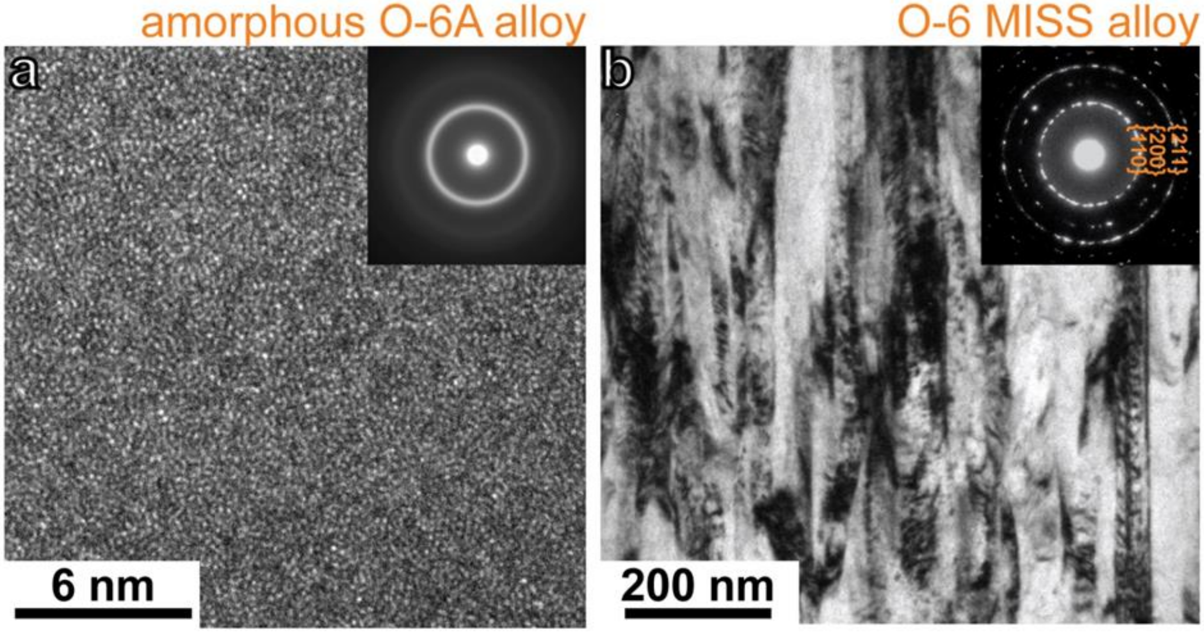


**Supplementary Fig. 2 TEM observations of the amorphous O-6A alloy and crystalline O-6 MISS alloy. a** HRTEM image of the amorphous O-6A alloy. A typical maze-like pattern in the HRTEM image and halo ring in the inserted SAED pattern indicate an amorphous structure of the alloy. **b** Typical side-view TEM image of the crystalline O-6 MISS alloy and the corresponding inserted SAED pattern.

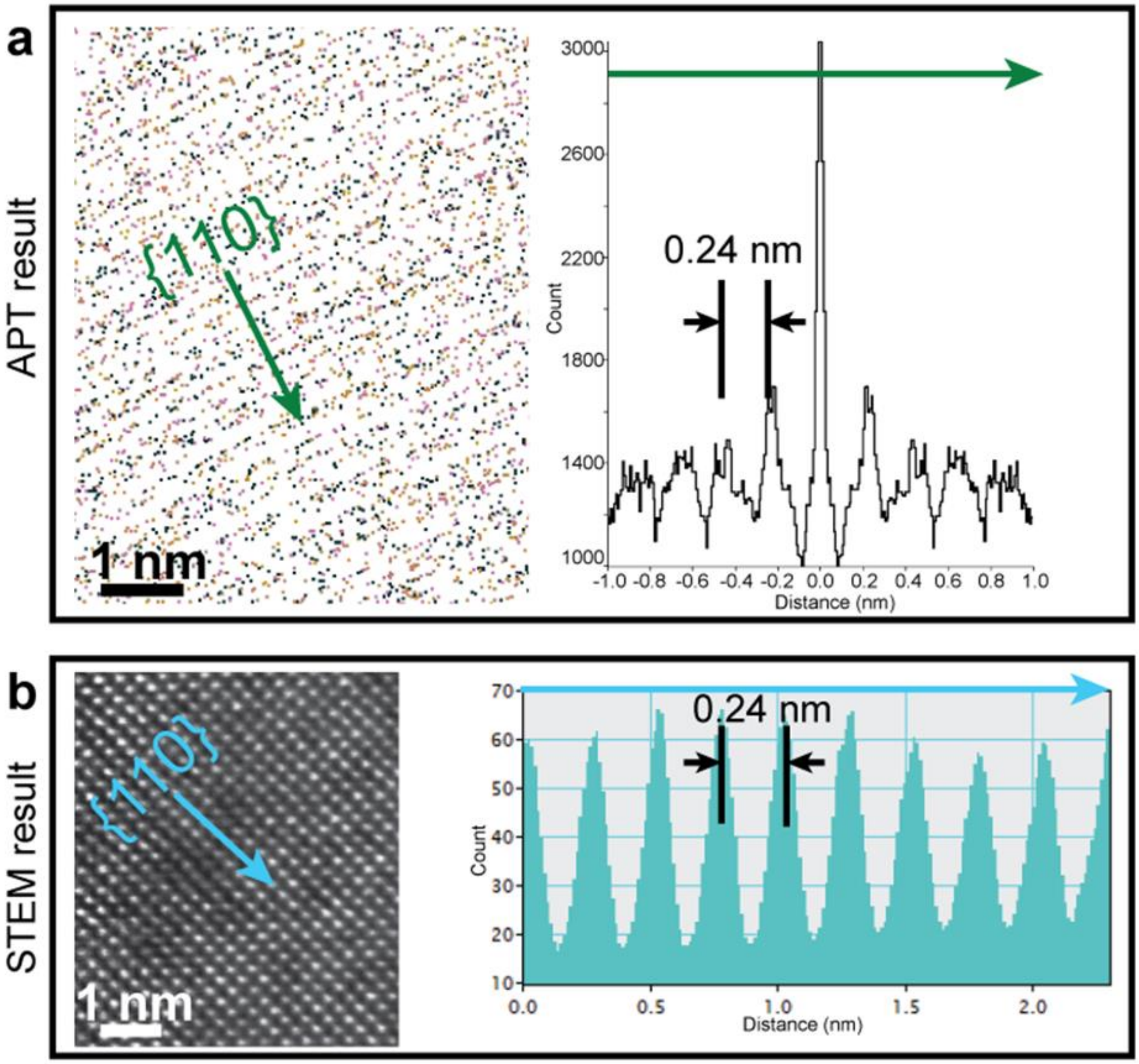


**Supplementary Fig. 3 APT and STEM investigations of the O-12 MISS alloy. a** Thin slice through the APT specimen presenting {110} atomic planes, the lattice spacing is measured to be 0.24 nm. **b** Atomic resolution STEM image showing the {110} atomic planes with a lattice spacing of 0.24 nm, which corresponds well with the APT observation.

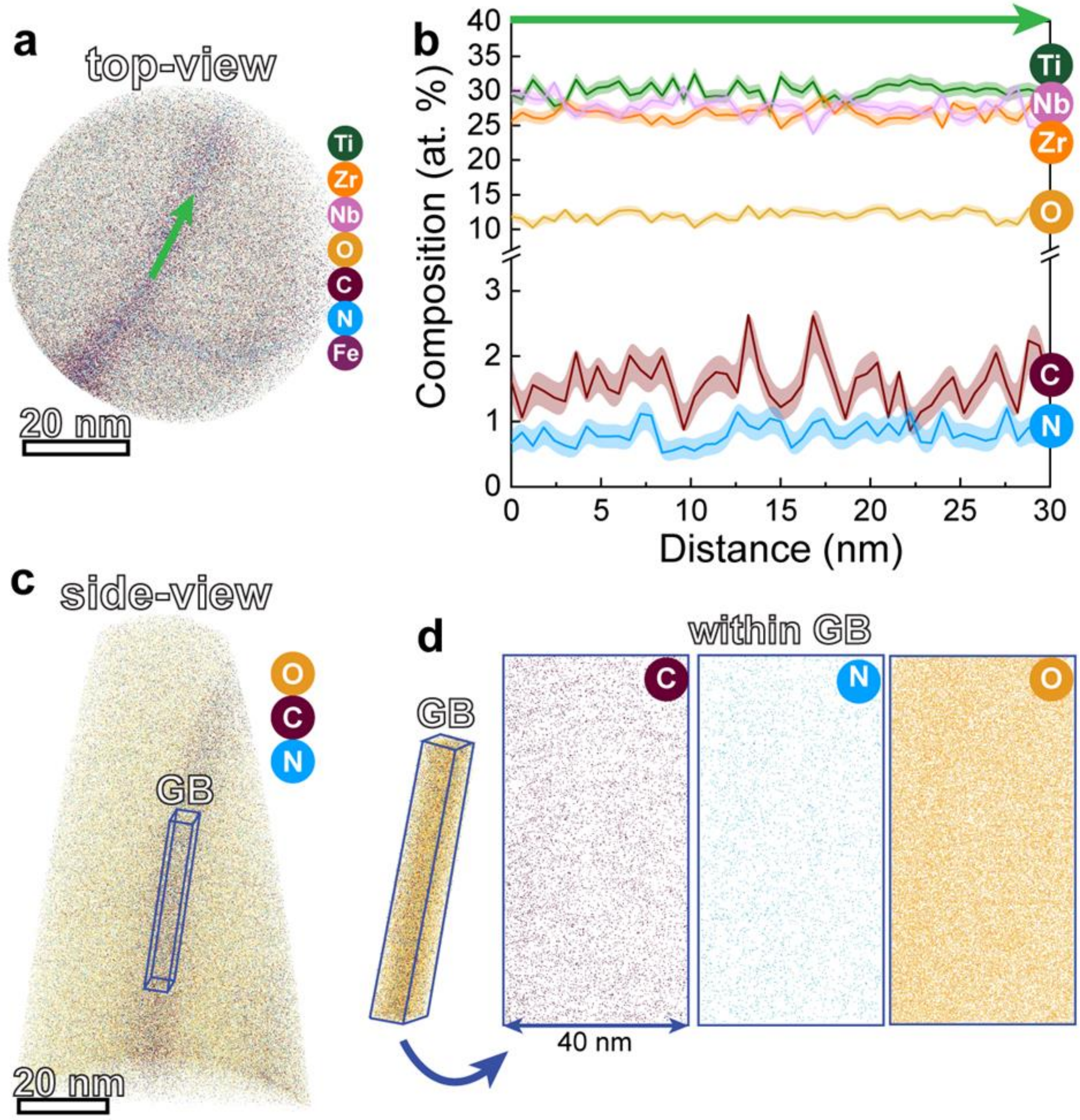


**Supplementary Fig. 4 APT analysis of a typical grain boundary in the O-12 MISS alloy. a** Top-view 30-nm-thick atom map from the three-dimensional reconstruction of an APT dataset, revealing that the grain boundaries are enriched with C and N. **b** 1D compositional profile measured along the grain boundary denoted by the green arrow in (**a**). The average contents of C and N in the grain boundary region are 1.8±0.3 at.% and 1.0±0.2 at.%, respectively. **c** Side-view of the three-dimensional reconstruction of the APT dataset. **d** Distribution of C, N, and O within

the grain boundary plane extracted from the blue cuboid in (**c**). C, N, and O show random distribution within the grain boundary plane, instead of clustering, confirming the absence of oxides, carbides, or nitrides in the MISS alloy.

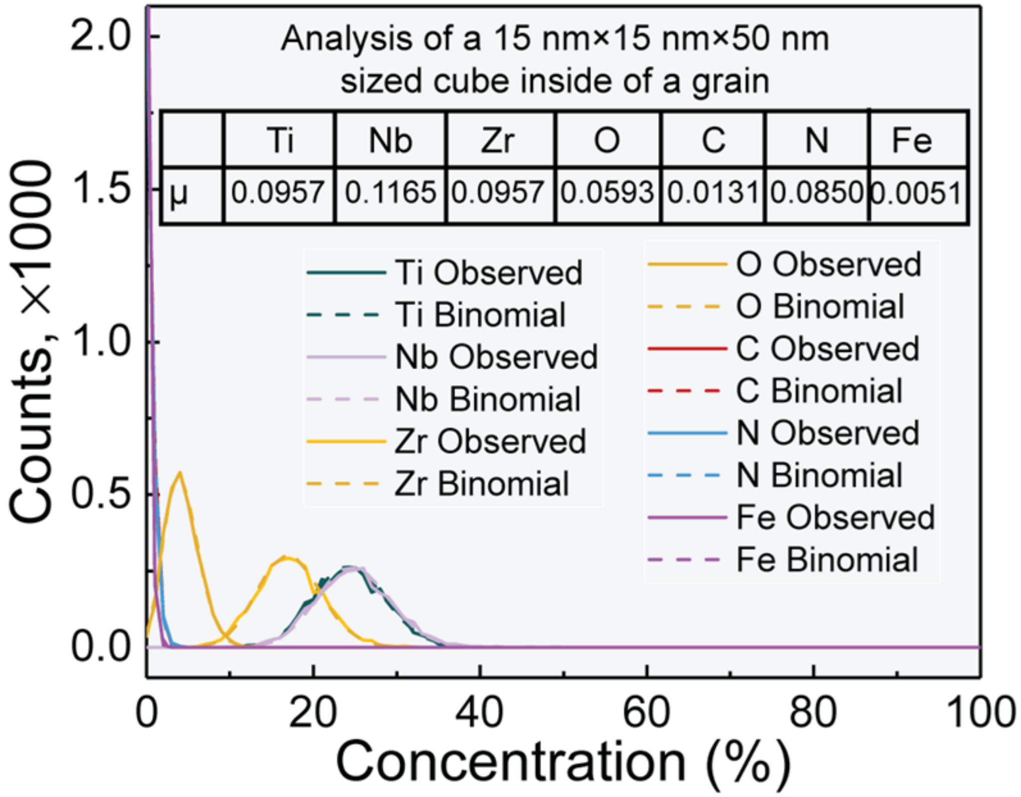


**Supplementary Fig. 5 Frequency distribution analysis of the elements inside of a grain in a typical APT dataset of the O-12 MISS alloy.** The existence of chemical short-range order[1] or concentration fluctuation[2] in certain alloys could affect dislocation motion and cross-slip. To demonstrate whether such chemical ordering effects exist in the current O-12 alloy, we performed an ion frequency distribution analysis (FDA) on the O-12 APT dataset. The distribution frequency of each element observed in experiment (solid lines) corroborates with the randomized dataset (dashed lines). Additionally, the Pearson coefficient μ for all elements is close to 0. These observations indicate a uniform distribution of all elements in nano- and near-atomic scales inside the nanograins.

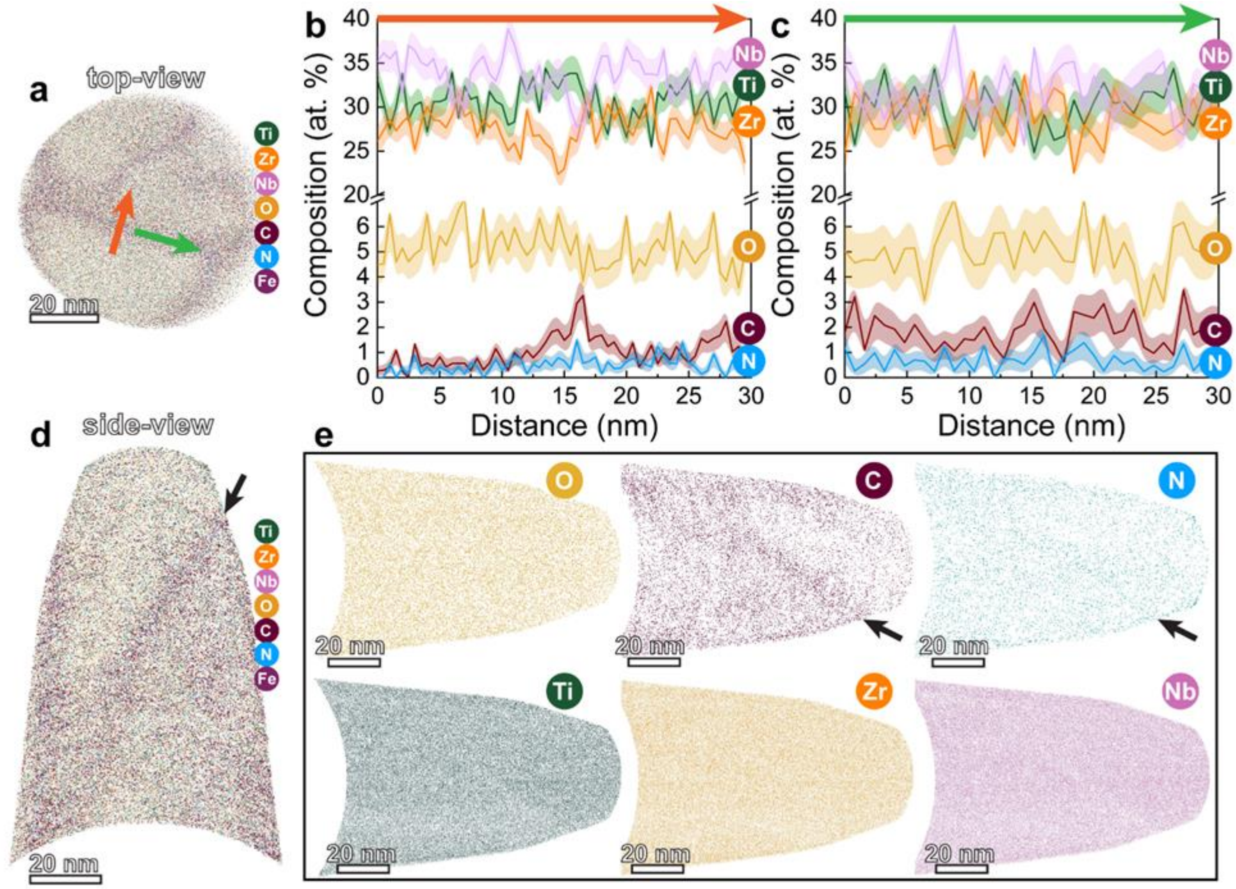


**Supplementary Fig. 6 APT analysis of the O-6 MISS alloy. a** Top-view 30-nm-thick atom map from the three-dimensional reconstruction of an APT dataset. The O-6 alloy has an average composition of $Ti_{31.1}Nb_{34.2}Zr_{27.3}O_{5.7}C_{0.9}N_{0.5}Fe_{0.3}$ (at. %), in which O distributes randomly while C and N are enriched at the grain boundaries. The green arrow marks the position of a grain boundary. **b** and **c** 1D compositional profiles measured along the orange (**b**) and green (**c**) arrows in (**a**). The average contents of C and N in the grain boundary region are 1.6±0.5 at.% and 0.8±0.3 at.%, respectively. **d** and **e** Side-view 10-nm-thick atom maps from the three-dimensional reconstruction of the APT dataset, confirming the absence of O, C, or N enriched clusters, i.e., oxides, carbides, or nitrides are not observed in this alloy.

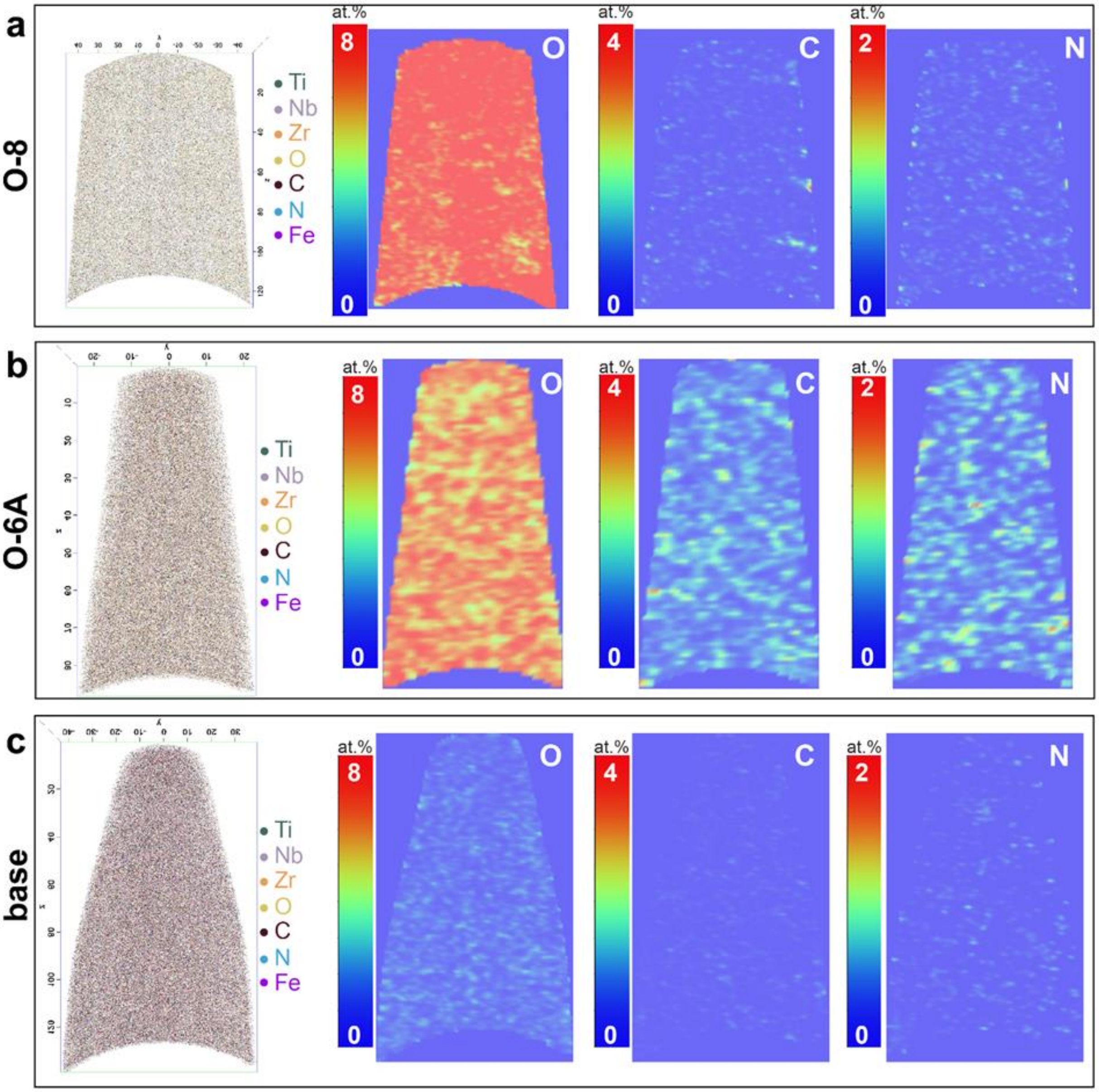


**Supplementary Fig. 7 APT characterization of the O-8, O-6A and base alloys. Left: side-view (10 nm-thick slice) of the APT specimens; right: 2D compositional maps (2-nm-thick slice).** **a** O distributes randomly in the O-8 MISS alloy. Moreover, the content of C and N is rather low, similar to that of the base alloy. **b** The amorphous O-6A alloy exhibits random elemental distributions. **c** The equiatomic TiNbZr base alloy consists of a negligible O, C, or N content,

confirming that neither the sputtering background vacuum nor the interstitial free alloy target introduces O/C/N elements into the alloy films.

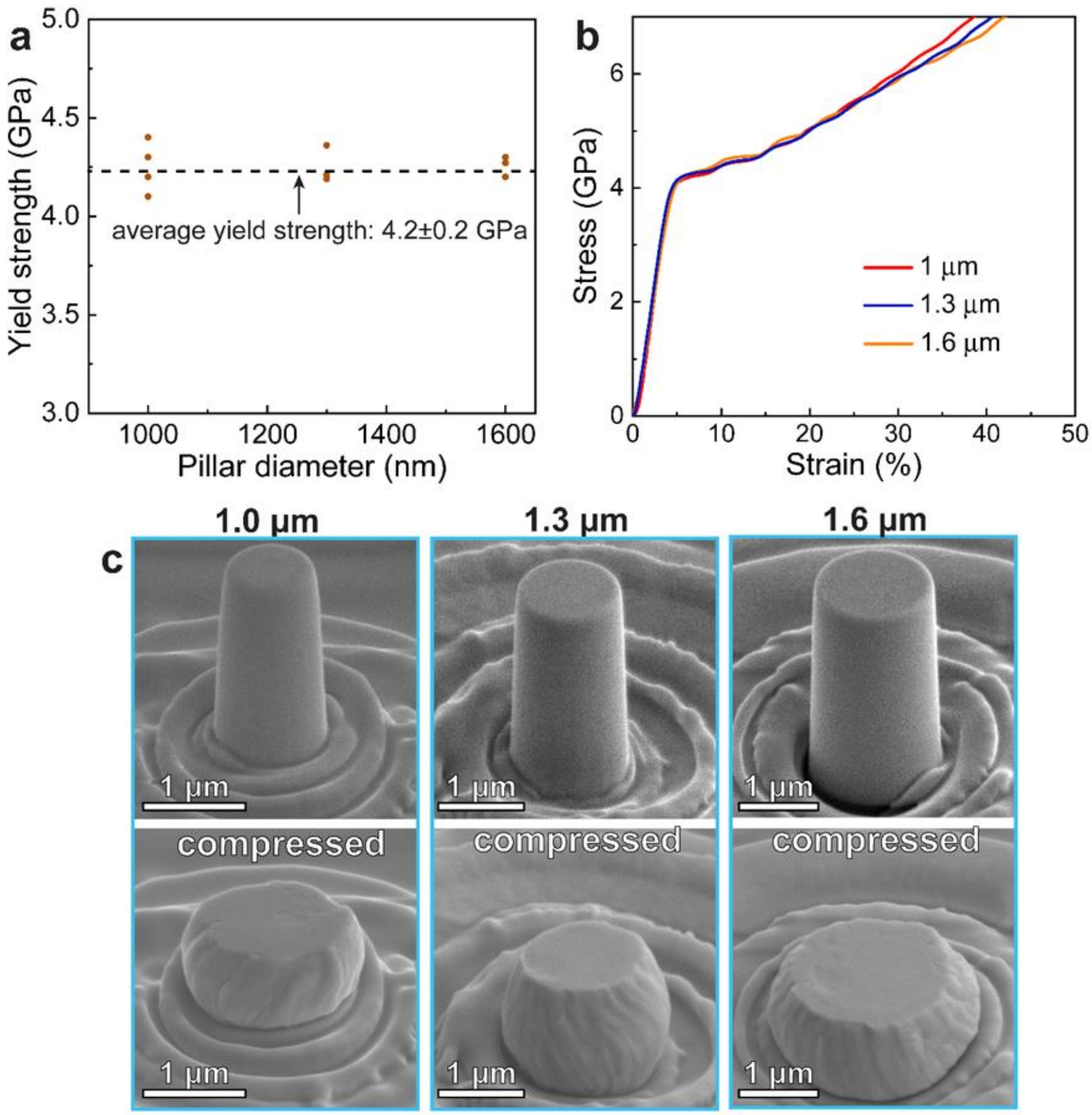


**Supplementary Fig. 8 Mechanical properties of the typical O-12 pillars with diameters of 1.0 μm, 1.3 μm, and 1.6 μm.** At least three pillars were tested for each dimension to ensure repeatability. **a** Yield strength of the pillars. The pillars with diameters ranging from 1.0 μm to 1.6 μm have the same average yield strength of 4.2 GPa. **b** Typical compressive engineering stress-strain curves of the pillars. **c** SEM images presenting the morphology of the pillars before and after compression. The pillars reveal homogeneous compressive deformation, as confirmed by the stress-strain curves (**b**) and SEM images (**c**).

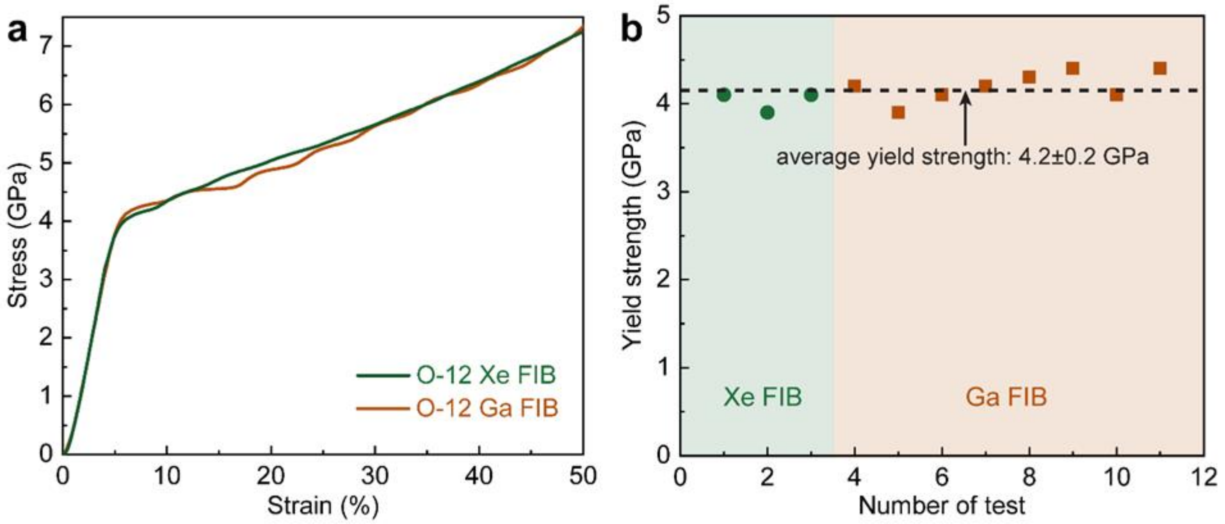


**Supplementary Fig. 9 Mechanical properties of O-12 micropillars prepared using Xe FIB and Ga FIB.** The pillars have the same diameters of 1 μm and the test conditions were identical. **a** Typical compressive engineering stress-strain curves of the O-12 pillars. **b** Yield strength of the pillars. Pillars fabricated using Xe and Ga FIB exhibit similar yield strength with an average value of 4.2±0.2 GPa. The stress-strain curve of the O-12 pillar fabricated by using Ga FIB reveals small steps in the plastic part, which is different from that prepared by Xe FIB. Ga is more electroactive and has a larger atomic radius than Xe, resulting in a stronger locking effect on dislocation motion, which promotes plastic instability of the alloy[3]. Besides, the O-12 alloy has an ultrahigh strength, in which the massive amounts of interstitials effectively retard the activation and motion of dislocations, hence, the $Ga^+$ locking effect on the surface of the pillars is more pronounced in the O-12 alloy compared to that in the O-6 and base alloys with lower strength.

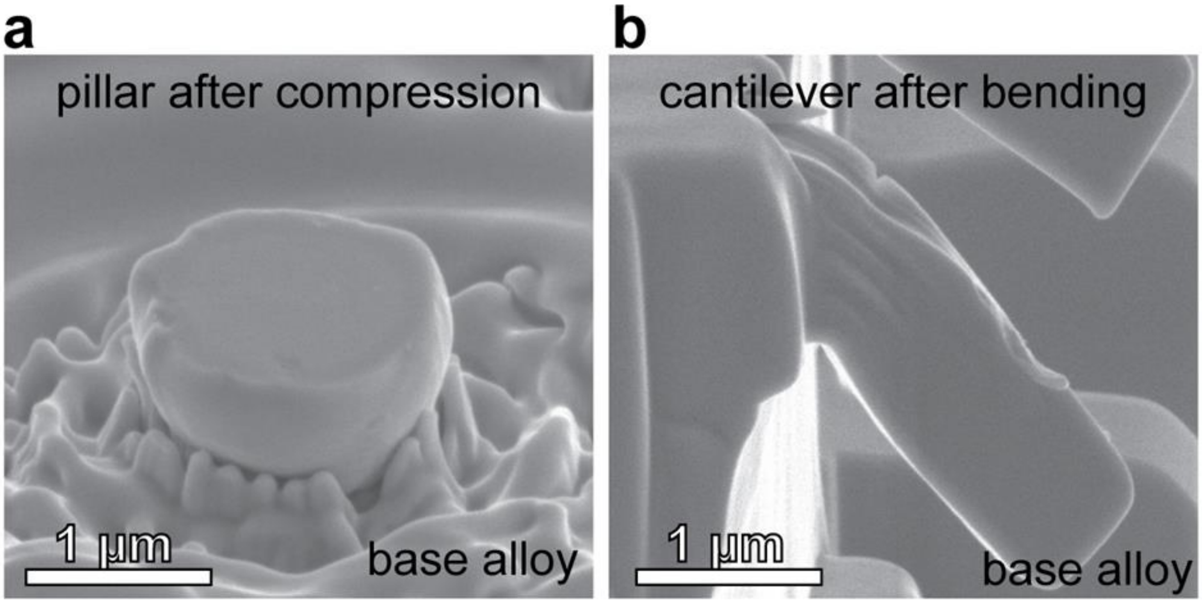


**Supplementary Fig. 10 SEM images presenting the morphology of the deformed base alloy.**

**a** Base alloy micropillar after compression, showing homogeneous deformation to a strain of 65%.

**b** Base alloy cantilever after bending, showing necking in the deformation region.

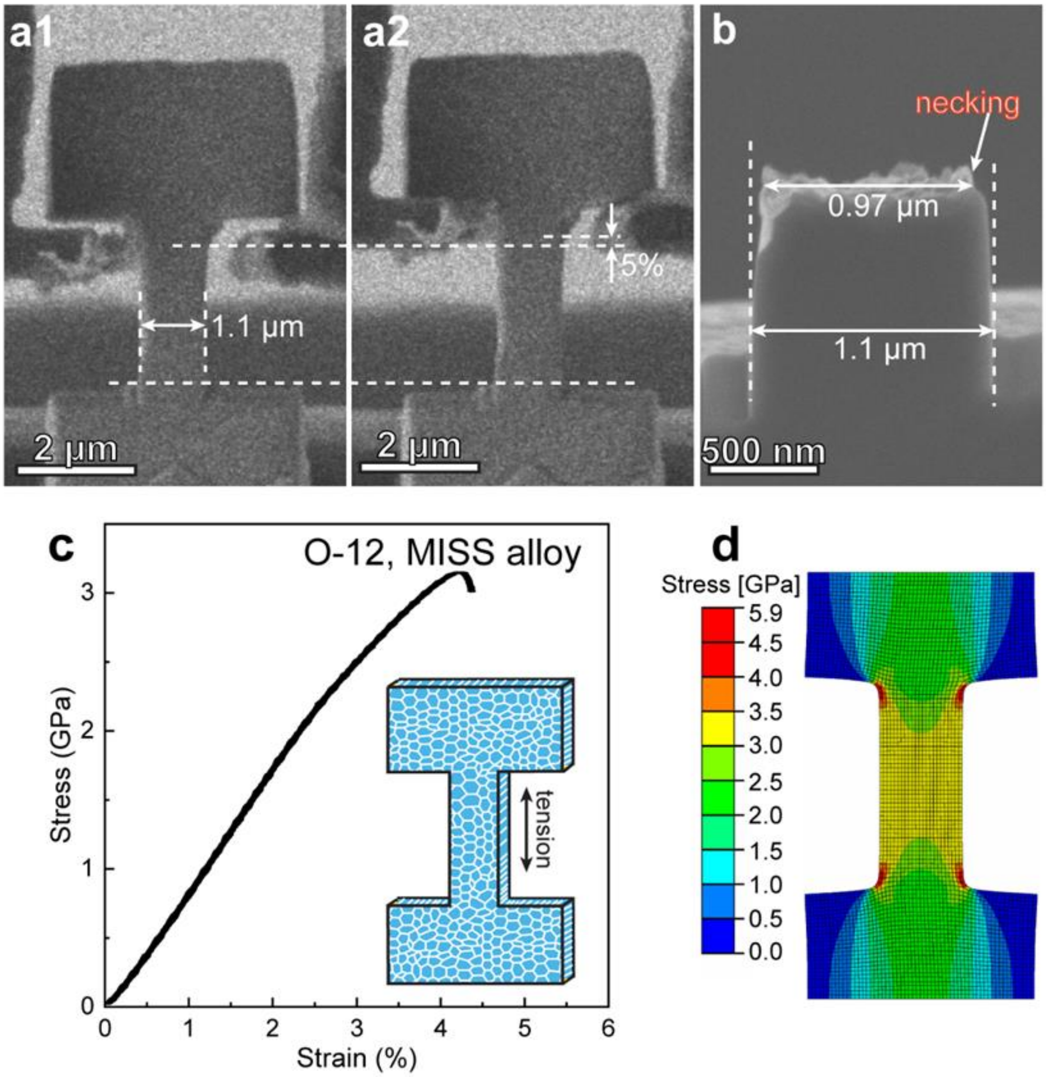


**Supplementary Fig. 11 Mechanical properties of the O-12 MISS alloy probed by an *in-situ* SEM tensile experiment. a** Snapshots from an *in-situ* SEM movie of the material's behavior under tension, presenting the morphology of the O-12 sample (**a1**) before tension and (**a2**) subjected to 5% tensile strain. **b** SEM image highlighting the necked region of the O-12 alloy after tension, which is a ductility demonstration for ultrastrong alloys in tension[4]. **c** Engineering stress-strain curve shows that the O-12 alloy withstands a tensile stress as high as 3.2 GPa, corresponding to a shear strength of 1.6 GPa. Considering that the O-12 alloy has a shear modulus of 38 GPa, the shear modulus-to-shear strength value is 24, approaching the theoretical strength regime. The inset

image schematically illustrates the loading direction relative to the orientation of the columnar grains. **d** FEM simulation of the stress distribution during tension. The FEM analysis reveals the occurrence of stress concentrations at the corners of the tensile sample, which can be ascribed to the relatively sharp geometries of the corners. We note a relatively uniform stress distribution at the middle part of the gauge section, with an estimated local tensile stress of ~3.2 GPa from the simulation. The simulation of the tensile stresses of the tested sample matches well with the experimental results. The FEM analysis confirms that the flow stress in tension is mainly determined by the middle part of the gauge section.

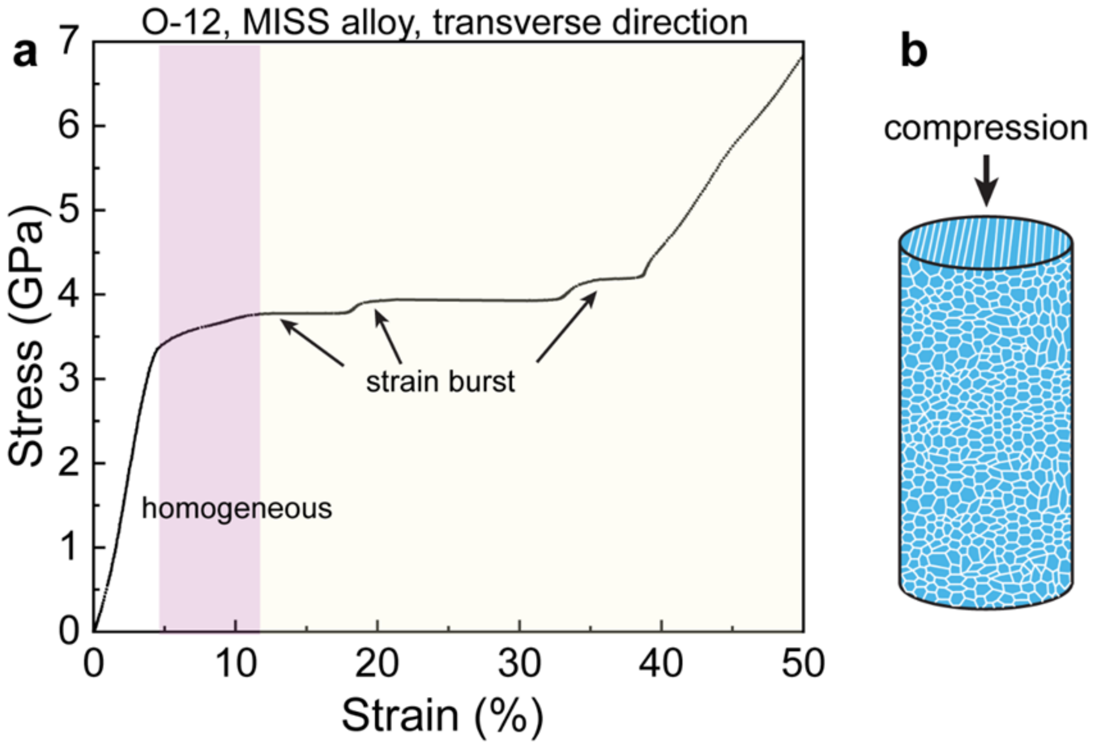


**Supplementary Fig. 12 Mechanical properties of the O-12 MISS alloy along the transverse direction. a** Engineering stress-strain curve from micropillar compression. The mechanical properties of the O-12 alloy in transverse orientation are also excellent, showing a yield strength of 3.4 GPa and homogeneous deformation to a strain of 12%. **b** Schematic diagram illustrating the compression direction with respect to the orientation of the columnar grains.

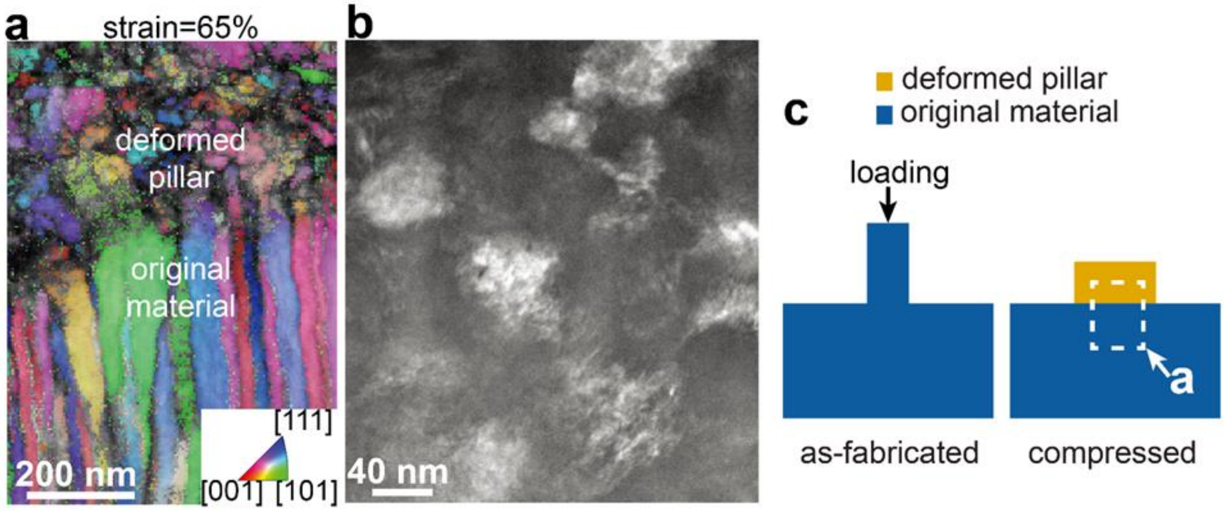


**Supplementary Fig. 13 Structure of the O-6 MISS alloy deformed to 65% strain. a** A side-view grain orientation map of the O-6 MISS alloy upon ~65% strain, presenting globular grains in the deformed region (upper part) and columnar grains in the original material (lower part). This grain orientation map was acquired using transmission Kikuchi diffraction (TKD). **b** A low-angle annular dark-field STEM (LAADF-STEM) image showing the refined grains in the deformed region of (**a**), confirming that the 'new' grains are globular. **c** A schematic diagram illustrating the location where the TKD specimen (**a**) was taken from.

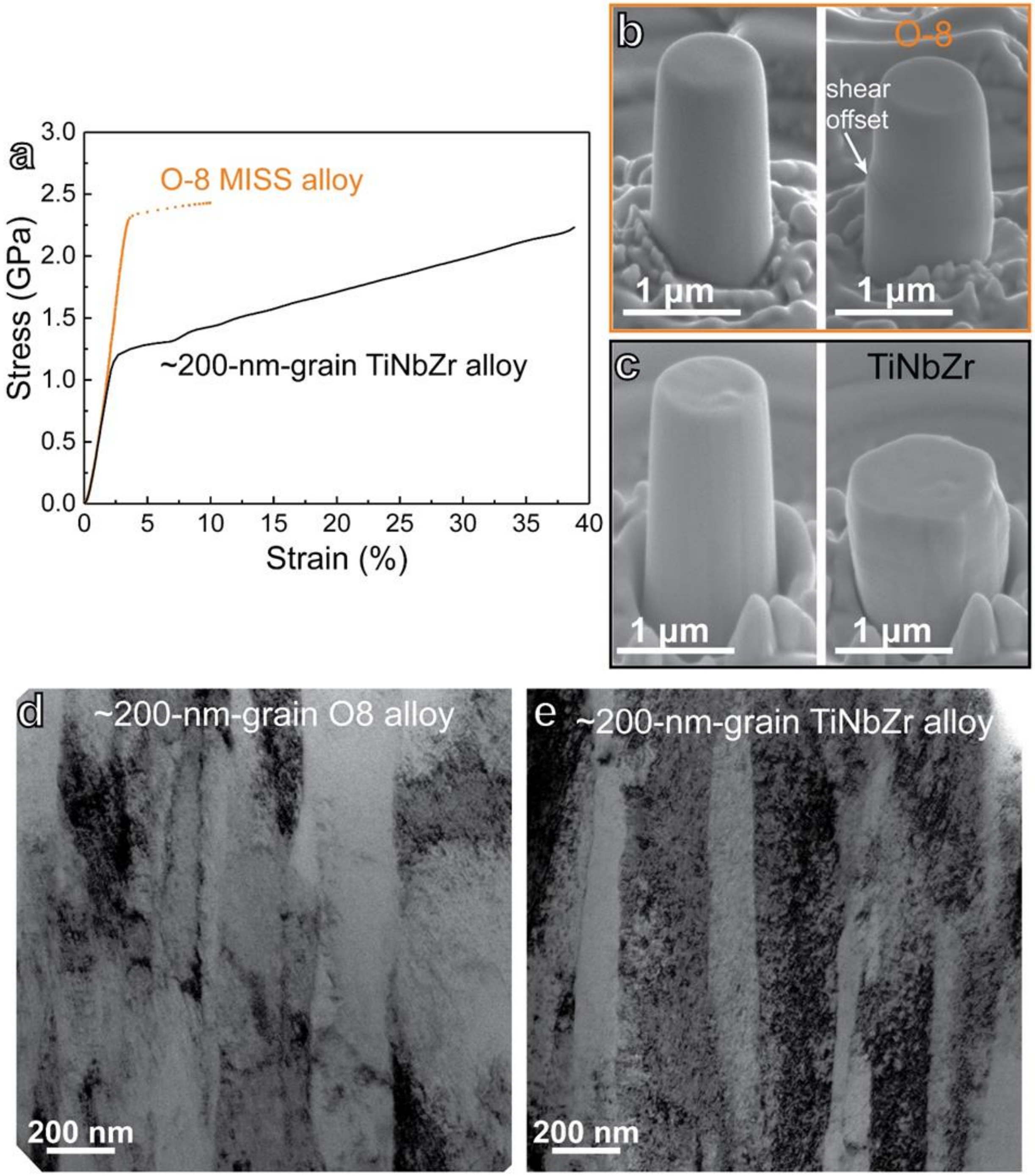


**Supplementary Fig. 14 Structure and mechanical properties of O-8 MISS alloy. a** Stress-strain curves of the O-8 MISS alloy and ~200-nm-grain TiNbZr alloy. **b** and **c** SEM images showing the O-8 MISS alloy (**b**) and TiNbZr alloy (**c**) pillar morphology before and after compression. **d** and **e** Typical side-view TEM images showing the columnar grains of the O-8 (**d**)

and TiNbZr (**e**) alloys. The O-8 MISS alloy was prepared by annealing the TiNbZr alloy at 500 $^{o}$C and $10^{-2}$ Pa for 2 hours, which results in a columnar grain size of ~200 nm. For comparison, we encapsulated the TiNbZr alloy in quartz tube which was evacuated ($<10^{-4}$ Pa) and then filled with Ar, and annealed this alloy at 500 $^{o}$C for 2 hours. The annealed TiNbZr alloy reveals a similar columnar grain size (~200 nm) with that of the O-8 alloy, serving as a reference data to evaluate the effect of O interstitials on the O-8 alloy.

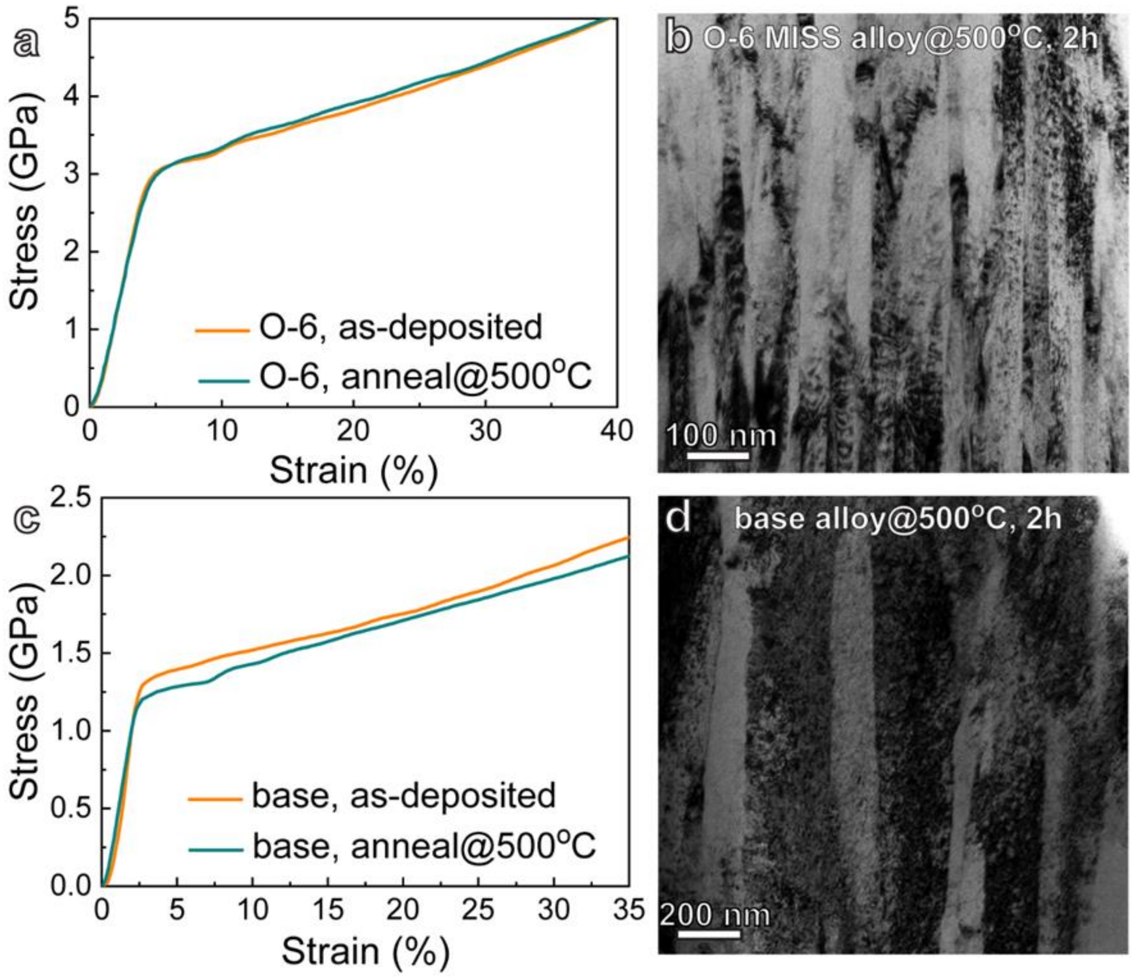


**Supplementary Fig. 15 Mechanical properties of the O-6 and base alloys after annealing at 500 °C for 2 hours.** The O-6 and base alloys were encapsulated in quartz tubes before annealing, and the tubes were evacuated (<$10^{-4}$ Pa) first and then filled with Ar. **a** Compressive engineering stress-strain curves of the as-deposited and annealed O-6 micropillars (1 μm diameter). **b** Typical side-view TEM image of the annealed O-6 alloy, showing that the columnar grain size is ~40 nm. **c** Stress-strain curves of the 1-μm-diameter micropillars fabricated from the as-deposited and annealed TiNbZr base alloys. **d** Side-view TEM image of the annealed base alloy, presenting a columnar grain size of ~200 nm. The as-deposited and annealed O-6 alloys exhibit an identical $\sigma_y$ of 2.9 GPa, while the $\sigma_y$ of the TiNbZr base alloy decreases from 1.3 GPa to 1.1 GPa after annealing. As discussed above, the as-deposited O-6 alloy has relaxed and decoration-stabilized

grain boundaries, which remain stable upon annealing at 500 $^{o}$C for 2 hours. This is also verified by the unchanged columnar grain size after annealing. By contrast, the TiNbZr base alloy without grain boundary segregation experiences grain growth upon annealing, accompanied by a decrease in yield strength.

## Supplementary References


1. Lei, Z. *et al.* Enhanced strength and ductility in a high-entropy alloy via ordered oxygen complexes. *Nature* **563**, 546–550 (2018).
2. Ding, Q. *et al.* Tuning element distribution, structure and properties by composition in high-entropy alloys. *Nature* **574**, 223–227 (2019).
3. Liu, J. *et al.* Effect of ion irradiation introduced by focused ion-beam milling on the mechanical behaviour of sub-micron-sized samples. *Sci. Rep.* **10**, 1–8 (2020).
4. Pan, J., Ivanov, Y. P., Zhou, W. H., Li, Y. & Greer, A. L. Strain-hardening and suppression of shear-banding in rejuvenated bulk metallic glass. *Nature* **578**, 559–562 (2020).
